# A computational model of coronary arteries with in-stent restenosis coupling hemodynamics and pharmacokinetics with growth mechanics

**DOI:** 10.1038/s41598-025-22291-w

**Published:** 2025-11-10

**Authors:** Anna Ranno, Kiran Manjunatha, Thore Koritzius, Ivo Steinbrecher, Norbert Hosters, Maximilian Nachtsheim, Pakhwan Nilcham, Nicole Schaaps, Anne Turoni-Glitz, Janina Datz, Alexander Popp, Kevin Linka, Felix Vogt, Marek Behr

**Affiliations:** 1 Chair for Computational analysis of Technical Systems, RWTH Aachen University, Aachen, Germany; 2 Institute of Applied Mechanics, RWTH Aachen University, Aachen, Germany; 3 Institute for Mathematics and Computer-Based Simulation, University of the Bundeswehr Munich, Neubiberg, Germany; 4 Department of Internal Medicine I - Cardiology, Angiology and Intensive Care Medicine, Uniklinik RWTH Aachen, Aachen, Germany; 5 Institute for Computational Mechanics, Technical University of Munich, Garching, Germany; 6 Institute for Continuum and Material Mechanics, Hamburg University of Technology, Hamburg, Germany

**Keywords:** Cardiology, Computational biology and bioinformatics, Engineering, Medical research

## Abstract

Despite advances in stent technology, in-stent restenosis remains a critical challenge following percutaneous coronary intervention. In this work, we propose a comprehensive fluid-solid computational model to simulate restenosis after drug-eluting stent implantation. We develop a three-dimensional continuum-based framework that couples the complex interplay of hemodynamics, pharmacokinetics, and restenosis-induced arterial growth. Within the arterial wall, a continuum model of cell dynamics and tissue growth predicts neointimal thickening. Drug release is modeled by direct diffusion from the abluminal stent surface and one-way absorption of hydrophobic drug from the bloodstream at the lumen-wall interface. We incorporate blood flow influence into growth mechanics through the effect of non-physiological wall shear stresses on endothelial cells morphology. Due to the short time scale inherent in the fluid model, we adopt a quasi-steady approach that efficiently homogenizes hemodynamic-related quantities over clinically relevant time scales for restenosis and drug release. We verify the components of the computational model and the quasi-steady assumption using a test case with an idealized cylindrical artery and a one-ring stent. The framework is further extended to patient-specific geometries obtained from optical coherence tomography and virtual stent implantation. Our results showcase how stent design, drug elution, and hemodynamics can collectively modulate restenosis progression, and the proposed coupling framework could, in the long term, contribute to the development of clinical decision-support tools.

## Introduction

Coronary artery disease, primarily caused by atherosclerosis, often necessitates percutaneous coronary intervention (PCI) to reopen narrowed vessels caused by atherosclerotic plaque. In many cases, this procedure involves the placement of an intravascular stent to restore blood flow to the heart. Due to the abrasion between the stent and the artery wall during a stent implantation procedure, the intima layer undergoes *endothelial denudation*. Thus, the luminal side of the vessel wall is stripped of the protection that the endothelial cells offer against potentially harmful components in the blood flow^1^. In addition, the interventional procedure is associated with vessel overstretch injuries such as rupture of internal and external elastic laminae^2^. Both triggers mentioned above initiate signaling events leading to pathological thickening of the vessel wall, gradually narrowing the artery and reducing blood flow – a condition known as in-stent restenosis (ISR). This overall mechanism is called *neointimal hyperplasia*. To develop the mathematical model, we identify key mediators of ISR and formulate a concise pathophysiological hypothesis.

Endothelial cells (ECs) regulate local hemostasis by releasing nitric oxide and prostacyclin. Following endothelial denudation or deep vascular injuries, the antiplatelet function of nitric oxide and prostacyclin is reduced, resulting in platelet and fibrinogen deposition at the injury sites^3^. Upon platelet activation, platelet-derived growth factor (PDGF) and transforming growth factor (TGF)-$\beta$, key components of platelet $\alpha$-granules, are released into the subintimal space of the vessel wall. Additionally, the inflammatory state at the stent implantation site increases the expression of intercellular and vascular cellular adhesion molecules. Consequently, monocytes infiltrate the subendothelial space, interact with cellular constituents and enhance the PDGF^4,5^. PDGF is a strong mitogen and chemokine for vascular smooth muscle cells (SMCs), which proliferate and migrate towards the intimal layer of the vessel wall^6^.The migration is facilitated by the degradation of the extracellular matrix (ECM), mainly containing collagen, which normally provides support for mature SMCs to adhere to. Matrix metalloproteinases, secreted within the vessel wall in response to PDGF, further accelerate collagen breakdown. TGF-$\beta$ influences SMC proliferation in a concentration-dependent manner: at low concentrations, it enhances proliferation; at higher concentrations, it reduces PDGF receptors, which then acts as an antiproliferative factor. These processes continue until the integrity of the endothelial monolayer is restored, while significantly thickening the intimal layer leading to the occlusive restenotic pathophysiology. In addition to the patient-specific immune response, restenotic growth is strongly influenced by the hemodynamics in the vessel. Low or oscillatory wall shear stress ($\text{WSS}$) acting on the endothelium has been associated with increased ISR^7^. It is hypothesized that pathological $\text{WSS}$ affects EC morphology, resulting in a leaky tiling of the endothelial monolayer. A compromised monolayer is ineffective in reducing platelet activation and preventing subsequent processes leading to ISR^8^.

Modern drug-eluting stents (DESs) consist of metal struts with a polymer layer on the strut surface that can release antiproliferative and anti-inflammatory drugs into the vessel wall at the implantation site^9^. These drugs are mainly rapamycin-analogs (e.g., sirolimus, everolimus, zotarolimus, etc.), which bind to FK506(tacrolimus)-binding proteins, specifically FKBP12. When this complex is bound to the mammalian target of rapamycin (mTOR) protein, the cell cycle progression beyond the G1 phase is arrested, and hence the proliferation is suppressed^10^. The drugs additionally inhibit the acute inflammatory response, reducing the expression of intercellular adhesion and vascular cellular adhesion molecules. Thus, the recruitment of monocytes into the subendothelial space is also limited. Despite reductions in ISR thanks to DESs, a significant portion of patients in clinical registries still exhibit restenosis^11^. This occurs because rapamycin-analogs indiscriminately target both smooth muscle cells and endothelial cells, delaying re-endothelization of the luminal surface. Consequently, the inflammatory response persists longer than with bare-metal stents.

A high-fidelity *in silico* replication of the complex pathophysiological process described above necessitates the development of a multiphysics framework tracking multiple constituents. In this context, several works have proposed employing coupled cellular automata and agent-based modeling strategies to describe the cellular processes^12–14^. On the other hand, finite element based hemodynamic evaluation coupled to agent-based models of ISR have also been presented^15,16^. Purely continuum-based descriptions, considering arterial overstretch achieved during stent implantation to be the main driver for growth, have additionally demonstrated reliable replicability of the restenotic process^17,18^. The volumetric growth has been accounted for via either the direct prescription of growth kinematics^19^, or constrained mixture models^20^, or the homogenized constrained mixture theory^21^. Recently, a hybrid continuum agent-based model has been presented to account for complex topological changes during ISR^22^.

A natural extension to the aforementioned models is the incorporation of pharmacological effects due to the drugs loaded onto DESs. Strategies have been extensively investigated^23–25^. For instance, the influence of different polymer coatings^26^, and drug binding through distinct species (e.g., bound and unbound drug) have been reviewed^27^. However, there is little consensus on the impact of drug released into the bloodstream. Some studies argue that the highly advective nature of blood flow washes out most of the luminally released drug^28^, whereas others find that this portion can significantly affect local drug distribution and tissue absorption^29^. Moreover, drug elution strongly depends on the drug properties – i.e., hydrophobic or hydrophilic – and its loading configuration on the stent (e.g., higher abluminal loading). While some research on hydrophobic drug transport in the arterial wall is available^30,31^, fewer studies address hydrophobic drug behavior in the blood.

Hemodynamics in coronary arteries has been investigated in previous studies, particularly for modeling patient-specific flow^32^. The presence of a stent can significantly disturb local hemodynamics, creating recirculation and requiring high-fidelity approaches for detailed resolution. Such efforts often focus on $\text{WSS}$-based indicators, but the computational cost rises due to the fine stent geometry in a relatively large coronary artery and the need for more advanced blood rheology models (e.g., shear-thinning^33^). Moreover, because arteries pulsate, fluid-structure interaction may be necessary to account for vessel motion^34^. When using a time-depending fluid domain with a boundary-conforming method to compute the flow field, the computational mesh has to be updated to account for the movement and to ensure a sufficient mesh quality. A popular method is the linear elastic mesh update method (EMUM). EMUM assumes the mesh to behave like a linear elastic solid^35^. The displacement of the mesh vertices is determined by solving the elasticity equation with the appropriate boundary conditions. It is widely used in simulations of biomedical applications on moving domains, particularly for arterial flow applications^36–40^.

In phenomena such as ISR, the slow boundary motion leads to tissue growth and remodeling over long time scales, while other processes, such as blood flow, occur on much faster scales. Various studies have addressed vascular growth and remodeling^41–45^, highlighting the complex interplay of mechanical properties, cell activity, and tissue adaptation. Solving this multiphysics problem with partitioned approaches is beneficial because existing solver frameworks can be re-used, limiting the implementation effort to coupling. Strongly coupled approaches with full convergence at each time step would yield the best accuracy^46^. However, solving the problem with the smallest characteristic time step in the system would lead to excessive simulation times. Instead, one can exploit the multiscale nature of the problem and apply specialized time-stepping techniques. A widely adopted strategy in the fluid-structure interaction community is the use of loose coupling for different time scales – for example, in modeling fibrin deposition in flow diverters^47^. Recent work has further explored managing multiphysics phenomena with disparate time scales in which one of the models reaches a periodic or quasi-steady regime, thereby allowing for *barely* coupled algorithms^48^.

Beyond the inherent modeling challenges, clinical relevance requires anatomically accurate representations of coronary arteries^49^, which can be obtained from imaging such as optical coherence tomography (OCT). OCT is a well-established high-resolution intravascular imaging modality that uses interferometry with short-coherence length light to visualize the luminal surface of coronary arteries at micrometer-level depth resolution^50^. As OCT provides a clear interface between the lumen and the intimal surface, it has emerged as a valuable diagnostic tool for better understanding the anatomical characteristics of coronary artery disease^51^. Moreover, it has been used to guide PCI, alongside the gold standard, angiography. We used OCT to optimize stent implantation and to evaluate whether additional PCI optimization was necessary. A further challenge arises in properly embedding the stent within the artery computational domain, given the stent geometrical complexity. Recently, a virtual stent implantation framework, based on mixed-dimensional modeling, has been presented^52^. Here, the stent struts are modelled using geometrically exact beam finite elements^53^. This requires mixed-dimensional coupling formulations^54–60^ to model the interaction between the beams and the artery. Among the most recent alternative approaches, applications to flow diverters have been presented^61^, and Pham et al.^62^ proposed a method for deriving stented configurations that is faster than full-physics finite element simulations. However, their approach relies on one-way coupling, assumes perfect stent expansion, and does not account for the material properties of the diseased vessel.

Many of the aspects required for a high-fidelity model of ISR have been explored in literature. However, modeling frameworks that account for the chemo-mechano-biological interactions involved in ISR – particularly those integrating hemodynamic feedback in the context of realistic, three-dimensional geometries – are scarce. The goal of this work is to develop a comprehensive modeling framework, by extending a previously established arterial wall model comprising of interactions between significant cell mediators, growth mechanics, and pharmacokinetics of rapamycin-analogs^63^. We investigate drug transport across the lumen-wall interface, including the modeling of hydrophobic drug release from drug-eluting stents. The influence of blood flow dynamics on the restenotic process is embedded via suitable hemodynamic indicators, while the lumen deformation caused by ISR growth is also reflected in the hemodynamics. Additionally, we reconcile the different time scales between hemodynamics and restenosic evolution through a quasi-steady strategy. We first test the coupling framework, along with the time homogenization assumption, using a simplified case of an idealized artery with a one-ring stent. We then adopt the virtual stent implantation as pre-processing step to generate the patient-specific geometry employed in the proposed coupling framework as proof-of-concept.

## Methods

**Figure 1:**
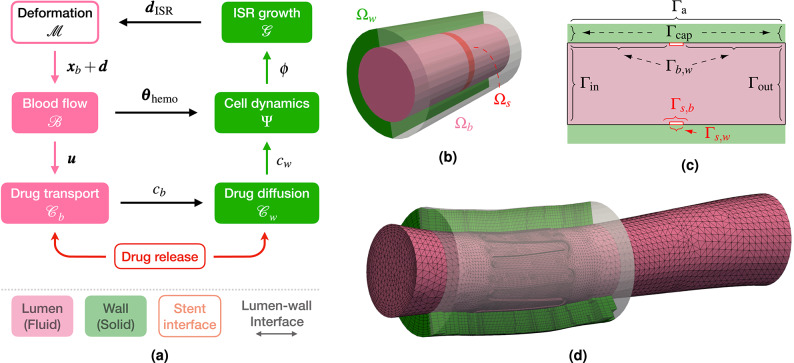
Computational model and domains. (**a**) Schematic representation of physical problems and their interaction, with legend (bottom row). The processes in the lumen are marked in pink, those in the artery wall in green, and the ones on the stent surface in red. The deformation problem ${\mathscr {M}}$ is marked differently because it is not a physical problem, but rather a pre-processing step, affecting the lumen geometry. The quantities exchanged at lumen-wall interface are driven by black arrows. (**b**) Fluid and solid domains for test case, with one-ring stent highlighted in red. (**c**) Fluid, solid, and interface boundaries on a longitudinal slice. (**d**) Fluid and solid meshes with matching interface nodes for patient-specific case with single-crown stent.

In this section, we clarify which physical interactions are included in our model and why others are neglected, particularly the structural interaction between the stent, blood, and artery wall. To model ISR in coronary arteries with DESs, multiple processes must be considered, as summarized in Fig. 1. Specifically, Fig. 1a is a skecth of the main physics and their interaction, representing only ISR-related processes occurring after PCI. Fig. 1b, c illustrate the corresponding domains and interfaces on the simplified test case of an idealized cylindrical artery segment with a ring-shaped stent.

To clarify the notation for boundaries, interfaces, and quantities, we denote the entire boundary of a domain $\Omega _*$ as $\Gamma _*$, and with $\bullet _*$ a physical quantity in that domain. Some processes occur exclusively in the artery wall $\Omega _w$ – solid domain – (shown in green), others in the artery lumen $\Omega _b$ – fluid domain – (in pink), and some are driven by the drug-eluting stent $\Omega _s$ (marked in red). When a variable is unambiguously associated with a specific domain (such as the blood velocity $\varvec{u}$ or cell dynamics $\phi$ in the wall) we omit the subscripts *b* and *w*, respectively. An interface shared by two domains $\Omega _*$ and $\Omega _{\dagger }$ is labeled $\Gamma _{*,\dagger }$. A boundary with a single subscript other than *b*, *w* or *s*, indicates that it belongs uniquely to one domain. In particular, $\Gamma _{\text {in}}$ and $\Gamma _{\text {out}}$ denote the inlet and outlet of the lumen domain $\Omega _b$, while $\Gamma _{\text {cap}}$ and $\Gamma _{\text {a}}$ correspond to the radial boundaries of the capped artery segment and to the outer adventitia of $\Omega _w$, respectively.

In the following paragraphs, we outline the ISR-related physics for a coronary artery (including lumen $\Omega _b$ and wall $\Omega _w$) with a DES, focusing on exchanges at the lumen-wall interface $\Gamma _{b,w}$ and at the stent interface $\Gamma _s$. In particular, we introduce several operators that represent the individual physical problems involved and motivate the simplified setup in Fig. 1a.

First, we note that the modeling framework starts after stent implantation. As no ISR-related processes occur in the stent volume $\Omega _s$, structural mechanics of the stent are not part of this Methods section. In the Results section, the contact mechanics of stent implantation between $\Omega _s$ and $\Omega _w$ are described only in the context of generating the more realistic geometry shown in Fig. 1d. Since no studies indicate that blood forces influence stent deployment, mechanics between $\Omega _s$ and $\Omega _b$ are not included. Instead, within this framework, we describe the implications of the presence of a DES for modeling the physics in $\Omega _b$ and $\Omega _w$. After deployment, the stent retains its shape and undergoes no deformation thereafter, effectively acting as a rigid body within the coronary artery.

We now describe the kinematic and dynamic coupling between the domains $\Omega _b$, $\Omega _s$, and $\Omega _w$, including, in particular, the tiles in Fig. 1a labeled “Blood Flow” and “ISR Growth”. In the fluid domain $\Omega _b$, blood flow ${\mathscr {B}}$ is modeled using Navier-Stokes equations, with velocity $\varvec{u}$ and pressure *p*, described in more detail in (2). The fluid shares interfaces with both the stent $\Omega _s$ and the artery wall $\Omega _w$, and thus, in principle, influenced by both. After deployment, the stent is rigidly embedded in the artery, acting only as an obstruction to the blood flow within the lumen. In the artery wall $\Omega _w$, we distinguish two sources of displacement: (i) compliant response to pulsatile blood pressure and (ii) displacement driven by ISR growth. In general, arterial walls exhibit pulsatile behavior that is modeled through fluid-structure interaction, for instance with a moving pressure wave through the artery^64^. This contribution is negligible in coronary arteries which are inherently stiff and further stiffened, locally, by metal stents^65^. Therefore, we assume that stresses – driven by the elastic response of the artery to blood pressure and potential electromechanical influences – are in equilibrium and neglect the compliant response. Hence, the blood pressure *p* does not play a pivotal role in our coupled model and is included only for completeness. The main source of displacement at the lumen-wall interface $\Gamma _{b,w}$ is, thus, due to ISR growth $\varvec{d}_{\text {ISR}}$. In the structural model of the wall domain $\Omega _w$, we consider the growth model ${\mathscr {G}}$ and solve for the displacement field $\varvec{d}_{\text {ISR}}$ from the balance of linear momentum and a hyperelastic constitutive law (see (11) for details). Notice that in this context, we do not consider the effect of arterial overstretch on the growth model for $\varvec{d}_\text {ISR}$. It is assumed to have a comparatively smaller impact on the restenotic process compared to the cell dynamics $\phi$, as already argued in a previous work^63^. The wall has common interfaces with both $\Omega _s$ and $\Omega _b$, but as discussed above, the stent is fixed and blood-induced loads are balanced. The kinematic and dynamic coupling conditions at the fluid-wall interface $\Gamma _{b,w}$, and the boundary conditions for the displacement field $\varvec{d}_{*}$, are summarized below:

$$\begin{aligned} \text {CC}_1: \qquad \left. \begin{array}{ll} \begin{aligned} & \varvec{d}_b = \varvec{d}_w:= \varvec{d}_{\text {ISR}} \quad \\ & \varvec{u}_b = \varvec{u}_w:= \varvec{u}_{\text {ISR}} \quad \\ & \varvec{\sigma }_{b}\varvec{n}_b = \varvec{\sigma }_{w}\varvec{n}_w \end{aligned} \end{array}\right\} \quad \text {on } \Gamma _{b,w}, \qquad \qquad \qquad \text {BC}_{\varvec{d}_{*}}: \qquad \begin{aligned}&\varvec{d}_{w} = \varvec{0} \quad && \text {on} \quad \Gamma _{\text {a}},\\&\varvec{d}_* \cdot \varvec{n}_* = 0 \quad && \text {on} \quad \Gamma _{\text {in}} \cup \Gamma _{\text {out}} \cup \Gamma _{\text {cap}},\\&\varvec{d}_{*} = \varvec{d}_{s} = \varvec{0} \quad && \text {on} \quad \Gamma _{s} = \Gamma _{s,b} \cup \Gamma _{s,w}, \end{aligned} \end{aligned}$$

with $\varvec{u}_*$ the velocity, $\varvec{\sigma }_*$ the stress tensor and $\varvec{n}_*$ the outward-facing unit normal vector. We explicitly keep the subscripts *b*, *w*, and *s* in the coupling conditions to emphasize that these apply across domain interfaces. We use a generic symbol $*$ in the boundary conditions to differentiate them from the notation $\varvec{d}$ in Fig. 1a and to indicate that the appropriate subscript should be inserted, depending on the boundary in question. Furthermore, the stent-wall and stent-blood interfaces are treated as rigid by prescribing homogeneous Dirichlet boundary conditions with $\varvec{d}_s = 0$.

Drug release from the stent surface $\Gamma _s$ is described by the two lower tiles in Fig. 1a: “Drug diffusion” into the artery wall $\Omega _w$ (green tile), and “Drug transport” into the lumen $\Omega _b$ (pink tile). The primary drug supply to the arterial wall stems from direct diffusion at the abluminal stent surface $\Gamma _{s,w}$. The total drug absorbed by the wall $c_w$ stems both from stent contact and downstream uptake from the blood, collectively modeled by the operator ${\mathscr {C}}_w$ (see (5) for details). On the fluid side, drug transport in the lumen is described by the operator ${\mathscr {C}}_b$ in (6). Hemodynamics governs drug release from the luminal stent side $\Gamma _{s,b}$ into the bloodstream. In particular, we couple blood velocity $\varvec{u}$ (one-way) to advect the drug $c_b$ downstream the artery. To correctly partition drug release across the stent surface $\Gamma _s$, we impose flux continuity at both interfaces with $\Omega _b$ and $\Omega _w$. Although the stent volume $\Omega _s$ is not explicitly modeled, this condition remains valid and is included here for completeness. In addition, drug flux across the lumen-wall interface $\Gamma _{b,w}$ is in equilibrium. The set of coupling and boundary conditions for the drug release problem is given below:

$$\begin{aligned} \text {CC}_{2}: \quad \begin{aligned}&\varvec{q}_{D} \cdot \varvec{n}_s = \varvec{q}_{c_b} \cdot \varvec{n}_b \quad && \text {on} \quad \Gamma _{s,b},\\&\varvec{q}_{D} \cdot \varvec{n}_s = \varvec{q}_{c_w} \cdot \varvec{n}_w \quad && \text {on} \quad \Gamma _{s,w},\\&\varvec{q}_{c_b} \cdot \varvec{n}_b = \varvec{q}_{c_w} \cdot \varvec{n}_w \quad && \text {on} \quad \Gamma _{b,w}, \end{aligned} \qquad \qquad \qquad \text {BC}_{c_*}: \quad \begin{aligned}&c_b = 0 \quad && \text {on} \quad \Gamma _{\text {in}},\\&\varvec{q}_{c_*} \cdot \varvec{n}_* = 0 \quad && \text {on} \quad \Gamma _{\text {out}} \cup \Gamma _{\text {a}} \cup \Gamma _{\text {cap}}, \end{aligned} \end{aligned}$$

where $\varvec{q}_{D}$ is the drug flux from the stent, and $\varvec{q}_{c_*}$ is the flux associated to the drug concentration $c_*$ for the corresponding domain $\Omega _*$. For the first two conditions in $\hbox {CC}_2$, we enforce a balance law to ensure that the total drug released from the stent surface $\Gamma _s = \Gamma _{s,b} \cup \Gamma _{s,w}$ equals the sum of drug fluxes into the lumen $\Omega _b$ and into the arterial wall $\Omega _w$. This balance is incorporated into the operators ${\mathscr {C}}_b$ and ${\mathscr {C}}_w$, through the boundary conditions at $\Gamma _{s,b}$ and $\Gamma _{s,w}$, respectively, as defined in (8). The third condition, on the lumen-wall interface $\Gamma _{b,w}$, is typically modeled as a Robin-type boundary condition, enforcing a drug flux proportional to the concentration difference $(c_b - c_w)$. Thus, drug moves from $\Omega _b$ to $\Omega _w$ when $c_b > c_w$, and vice versa when $c_w > c_b$^66^. In this work, we consider a hydrophobic drug, which exhibits low solubility in water, and thus also in blood. Instead, it adheres to cell-membrane-rich tissues, such as the artery wall. Consequently, we enforce a one-way flux of $c_b$ from the bloodstream into the wall, without allowing reverse diffusion back into the lumen.

The final physics component in the ISR framework is the “Cell dynamics” (green tile) in Fig. 1a. Within the solid domain, experimental evidence indicates that the mechanics of ISR growth ${\mathscr {G}}$ is mainly driven by SMCs infiltration into the intimal layer, forming a *neointima*. As this layer expands, it moves the original arterial wall configuration by the growth displacement $\varvec{d}_{\text {ISR}}$. The cell distributions $\phi$ (including SMCs) evolve via a system of interaction dynamics and coupled reactions summarized by $\Psi$ (see (9) for more details). In particular, SMCs proliferation depends strongly on PDGF and TGF, which are influenced by two main factors: (i) the anti-proliferative drug $c_w$ released from the stent, and (ii) the effect of hemodynamic indicators, represented by $\varvec{\theta }_{\text {hemo}}$ in (3), on ECs. Rapamycin-analog drugs inhibit ISR by suppressing excessive SMC proliferation but may also affect other cells as a side effect. To account for the effect of the drug $c_w$, the system of equations defined in $\Psi$ and ${\mathscr {C}}_w$ are solved monolithically. For further details on pharmacokinetics within the arterial wall, we refer to our previous work^63^. Regarding the effect of altered shear stresses, hemodynamics strongly influences the health of ECs and the artery wall. Prior studies indicate that recirculation, stagnation, and other non-physiological flow patterns – particularly those tied to $\text{WSS}$ – significantly impact EC physiology^7^. After stent implantation, endothelial injury leads to inflammation, platelet activation, and monocyte infiltration into the arterial wall, increasing PDGF and TGF levels. As new ECs form, the release of PDGF and TGF should ideally decrease. However, if disturbed blood flow persists, ECs assume non-physiological shapes, resulting in gaps that facilitate ongoing monocyte infiltration, sustaining inflammation and continuous PDGF and TGF production. This effect is incorporated into the cell dynamics model as an increased boundary flux of PDGF and TGF on the lumen-wall interface $\Gamma _{b,w}$, modulated by the hemodynamic indicators (see (10) for details). Conversely, the cell dynamics are fully contained within the solid domain $\Omega _w$ and do not influence the blood flow, resulting in a one-way coupling.

We can identify two distinct time scales between the fluid and the solid model: a macro time scale of weeks to months for ISR progression and drug release, denoted by $T$, and a micro time scale of seconds, denoted by $T_b$ for hemodynamics. To address this disparity, we employ a quasi-steady approach that captures the essential hemodynamic-related quantities without significant loss of information. In the next subsection, we introduce the coupling algorithm, where we describe how we manage the different time scales and which simplifications are introduced in the multiphysics formulation. The remainder of this section is organized as follows: after the coupling algorithm, we follow by detailed modeling of individual processes, including the homogenization and quasi-steady strategies where relevant, and conclude with the OCT segmentation procedure.

### Coupling algorithm and time scales

The general procedure to solve the coupled problems follows the sequentially staggered approach below. The operators ${\mathscr {M}}$, ${\mathscr {B}}$, ${\mathscr {C}}_b$, ${\mathscr {C}}_w$, $\Psi$, and ${\mathscr {G}}$ are used in the following sections to represent the individual problems, with their respective equations, initial and boundary conditions. Readers interested in the initial boundary value problem of each single field can refer directly to the corresponding equations that are explicitly marked in the algorithm.

Even if only surface information at the lumen-wall interface $\Gamma _{b,w}$ needs to be exchanged, the operators ${\mathscr {M}}$ and ${\mathscr {C}}_w$ are getting the complete field data of the coupled problems as input to simplify the notation further. To point out the mutual dependency of the fields involved, only external input of other fields is noted as input, i.e., the solution of each single field problem from previous time steps is considered as given. Consequently, the procedure is as follows:

**Figure:**
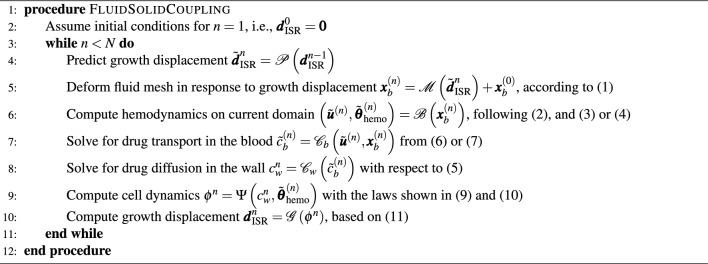

In the algorithm described above, the superscript $\bullet ^n$ indicates the corresponding time step for all unknowns, while the $\tilde{\bullet }$ marks a value predicted under certain assumptions, within iteration $\bullet ^{(n)}$, as introduced in the following.

For optimal accuracy, the coupling scheme should be driven by the smallest characteristic time scale of the fields involved. In the fluid model, the unsteady dynamics occur on a time scale $T_b$ comparable to an average heartbeat ($T_{HB}=0.83 ~\text{[s]}$), which requires an appropriate time step size of $\Delta t_b = {\mathscr {O}}(10^{-3})~\text {[s]}$. However, simulations of the full problem with time step size $\Delta t_b$ would lead to prohibitively long computation times, since the characteristic time horizon $T = N\Delta t_w$ for the drug release and ISR is in the scale of several weeks, as observed from experimental evidence^67^. Thus, as coupling step, we propose here the related intrinsic time step size $\Delta t_w = 1~\text {[day]}$, justified by previous convergence studies of the standalone solid model^68^. However, since this time step is too large to resolve all physical phenomena in the fluid, we propose an alternative solution, similarly to the *barely coupled* multiphysics approach proposed by Lohner et al.^48^. Under the assumption that the comparably slow dynamics of drug release and ISR do not significantly affect the fluid, we adopt a quasi-steady approach.

The main simplifications introduced by this approach are summarized in the following:

1. Due to very minor changes between two days, the ISR displacement is computed based on a zero-order predictor of the growth factor: ${\mathscr {P}}\left( {\varvec{d}}_{\text {ISR}}^{n-1}\right) = {\varvec{d}}_{\text {ISR}}^{n-1}$. Since the displacement evolves very slowly, we assume $\varvec{u}_\text {ISR}\approx \varvec{0}$ within $T_b$.
2. $\Omega _b = \Omega _b^{(n)}$ changes at every daily time iteration only and is assumed to be fixed within each $\Delta t_w$ interval.
3. We assume that the drug concentration $c_b$ is in equilibrium at the interface $\Gamma _{b,w}$ within $T_b$, and thus locally impose $\varvec{q}_{c_b} \cdot \varvec{n}_b = 0$.
4. On this fixed domain $\Omega _b$, the flow problem ${\mathscr {B}}$ and drug transport ${\mathscr {C}}_b$ are solved within each coupling step only as steady problems, which significantly enhances the efficiency of the procedure.

The first three assumptions further simplify the previously defined coupling conditions $\hbox {CC}_1$ and $\hbox {CC}_2$ and require the following adaptations:

$$\begin{aligned} \widetilde{\text {CC}}_1: \qquad \left. \begin{array}{ll} \begin{aligned} & \varvec{u}: = \varvec{u}_b = \varvec{0}\quad \\ & \varvec{\sigma }_{b}\varvec{n}_b = \varvec{\sigma }_{w}\varvec{n}_w \end{aligned} \end{array}\right\} \quad \text {on } \Gamma _{b,w}, \qquad \qquad \qquad \widetilde{\text {CC}}_{2}: \qquad \begin{aligned}&\text {[same as CC}_2\text {]} \quad && \text {on} \quad \Gamma _{s},\\&\varvec{q}_{c_b} \cdot \varvec{n}_b = 0 \quad && \text {on} \quad \Gamma _{b,w},\\&\varvec{q}_{c_w} \cdot \varvec{n}_w = F(c_b-c_w)\quad && \text {on} \quad \Gamma _{b,w}. \end{aligned} \end{aligned}$$

The key interaction between wall and lumen involves accounting for how ISR-induced wall deformation affects hemodynamics. Severe ISR with a substantial displacement $\varvec{d}_{\text {ISR}}$ can narrow the lumen, altering blood flow. However, due to the slow progression of growth, in $\widetilde{\text {CC}}_1$ we modify the original kinematic condition on $\varvec{u}_b$ and neglect the one for $\varvec{d}:= \varvec{d}_b$. Because of assumption 2, the fluid domain deformation ${\mathscr {M}}$ is not a physical component of the ISR process itself but rather a necessary pre-processing step for accurately updating the lumen geometry in our simulations (hence, it is sketched differently in Fig. 1a). This is computed via an elastic mesh update method in response to the wall displacement $\varvec{d}_{\text {ISR}}$ on $\Gamma _{b,w}$ (see (1) for details). Due to the slow time scale governing drug evolution in the arterial wall ${\mathscr {C}}_w$, the fluid model ${\mathscr {C}}_b$ cannot resolve the cumulative drug absorption at the lumen-wall interface $\Gamma _{b,w}$. Instead, we assume that over the short timescale $T_b$, drug concentration at the interface is in local equilibrium, i.e., $c_b = c_w$. In the adapted $\widetilde{\text {CC}}_2$, this leads to a Neumann-Robin coupling condition: a homogeneous Neumann boundary condition is imposed for ${\mathscr {C}}_b$ (see the corresponding line in (7)), while drug absorption in ${\mathscr {C}}_w$ is still imposed with a Robin boundary condition at $\Gamma _{b,w}$ (see the corresponding line in (5)). We verify our methodology and the quasi-steady assumption in Fig. 3 using the test case of an idealized artery segment with a ring stent. In particular, to verify the fourth simplification, the results are compared with unsteady simulations on the fixed fluid domains $\Omega _b^{(n)}$. Since the boundary conditions remain fixed within each coupling step $\Delta t_w$, the unsteady fluid simulation is computed only until a periodic regime is reached – typically after three heartbeats. In contrast to simplification 4, the required fluid quantities $\tilde{\varvec{u}}^{(n)}$, $\tilde{\varvec{\theta }}^{(n)}_{\text {hemo}}$ and $\tilde{c}^{(n)}_b$ are averaged over one cardiac cycle $\Delta T_{HB}$ and, then, incorporated into the remaining coupled problems (see Algorithm 1, lines 6 and 7). For more details on the test cases setup, we refer to Table 1.

### Elastic mesh update method for fluid domain deformation

In a boundary conforming discretization, the computational grid of the blood domain $\Omega _b(\varvec{x}_b)$ has to be adapted to any boundary displacement, i.e., to displacement $\varvec{d}_{\text {ISR}}$ on the lumen-wall interface $\Gamma _{b,w}$ caused by the ISR growth. Here, the needed displacement field $\varvec{d}$ of the grid nodes is computed by the linear elastic mesh update method with respect to the initial configuration on $\Omega _b^{(0)}=\Omega _b\left(\varvec{x}^{(0)}_b\right)$ and $\Gamma _b^{(0)} = \Gamma _b\left(\varvec{x}^{(0)}_b\right)$. The ISR growth is incorporated via Dirichlet conditions at the interface boundary $\Gamma _{b,w}^{(0)} = \Gamma _{b,w}\left(\varvec{x}^{(0)}_b\right)$, leading to the following system of equations:

1 $$\begin{aligned} {\mathscr {M}}(\varvec{d}_{\text {ISR}}): \qquad \begin{aligned}&\nabla \cdot \varvec{\sigma }_M(\varvec{d})=\varvec{0} \quad && \text {in} \quad \Omega ^{(0)}_b,\\&\varvec{d} = \varvec{d}_{\text {ISR}} \quad && \text {on} \quad \Gamma ^{(0)}_{b,w},\\&\varvec{d} = \varvec{0} \quad && \text {on} \quad \Gamma ^{(0)}_b \setminus \Gamma ^{(0)}_{b,w}. \end{aligned} \end{aligned}$$

The consitutive model to close Eq. (1) is defined by the stress-strain relation $\varvec{\sigma }_M(\varvec{d}) = \frac{\lambda _M}{\mu _M}\text {tr} \left( \varvec{E}_M(\varvec{d}) \right) \varvec{I} + 2\varvec{E}_M(\varvec{d})$, and the infinitesimal strain tensor $\varvec{E}_M(\varvec{d}) = \frac{1}{2} \left( \nabla \varvec{d} + \nabla \varvec{d}^\top \right)$. The Lamé parameters are chosen as $\mu _M = 1.0 ~[\text {kg/mm/s}^2]$, $\lambda _M = 1.0~[\text {kg/mm/s}^2].$ Algorithm 1 (lines 4 and 5) details the choice of $\varvec{d}_{\text {ISR}}$ and the update rule.

### Blood flow model and hemodynamic indicators

To model blood flow, we employ unsteady Navier-Stokes equations. We compute the velocity field $\varvec{u} = \varvec{u}(\varvec{x}_b)$ and pressure $p = p(\varvec{x}_b)$ within the fluid domain $\Omega _b = \Omega _b(\varvec{x}_b)$ over the characteristic time horizon of fluid phenomena $T_b$, from the momentum and mass balance:

2 $$\begin{aligned} {\mathscr {B}}(\varvec{x}_b): \qquad \begin{aligned}&\rho _b \left( \frac{\partial \varvec{u}}{\partial t}+\varvec{u}\cdot \nabla \varvec{u}\right) -\nabla \cdot \varvec{\sigma }_b = \varvec{0} \quad && \text {in} \quad \Omega _b \times (0, T_b),\\&\nabla \cdot \varvec{u} = 0,\quad && \text {in}\quad \Omega _b \times (0, T_b),\\&\varvec{u} = \varvec{0} \quad && \text {in} \quad \Omega _b \times \{ t = 0\},\\&\varvec{u} = \varvec{f}_{\text {in}} \quad && \text {on} \quad \Gamma_{\text {in}},\\&\varvec{u} = \varvec{0} \quad && \text {on} \quad \Gamma_{b,w} \cup \Gamma _{s,b},\\&\varvec{\sigma }_b~\varvec{n}_b= \varvec{0} \quad && \text {on} \quad \Gamma _{\text {out}}, \end{aligned} \end{aligned}$$

where $\rho _b = 1.058 \cdot 10^{-6}~[\text {kg/mm}^3]$ is the blood density, $\varvec{\sigma }_b$ represents the fluid stress tensor, and $\varvec{n}_b$ is the outward-facing unit normal. Blood is modeled as incompressible Newtonian fluid^33^ with $\varvec{\sigma }_b = -p \varvec{I} + 2 \mu _b \varvec{E}(\varvec{u})$, where $\varvec{E}(\varvec{u}) = \frac{1}{2} \left( \nabla \varvec{u} + \nabla \varvec{u}^\top \right)$ is the strain-rate tensor, and $\mu _b = 3.5 \cdot 10^{-6}~\text {[kg/mm/s]}$ is the dynamic viscosity. The inflow boundary condition $\varvec{f}_{\text {in}} = \varvec{f}_{\text {in}}(\varvec{x}_b, t)$ specifies a parabolic velocity profile derived from the time-dependent flow rate $Q(t)$, which is representative of an average right coronary artery flow^69^. The shear stress magnitude is computed as $\text {WSS}= \vert \varvec{\tau } \vert = \vert 2 \mu _b (\varvec{E} \varvec{n}_b - [(\varvec{E} \varvec{n}_b) \cdot \varvec{n}_b] \varvec{n}_b) \vert$, where $\varvec{\tau }$ represents the shear stress tensor. From the $\text{WSS}$, we derive the time-averaged hemodynamic indicators^70^ $\varvec{\theta }_{\text {hemo}} = \left( \text {TAWSS}, \text {OSI}\right)$:

3 $$\begin{aligned} \text {TAWSS}= \frac{1}{T_{b}}\int _0^{\Delta T_{b}} \text {WSS}~dt, \qquad \text {OSI}= \frac{1}{2}\left( 1-\frac{ \vert \int _0^{\Delta T_{b}} \varvec{\tau }~dt \vert }{\int _0^{\Delta T_{b}} \vert \varvec{\tau } \vert dt}\right) , \end{aligned}$$

where $\text {TAWSS}$ is the time-averaged wall shear stress, and $\text {OSI}$ is the oscillatory shear index.

Algorithm 1 (line 6) determines how the blood velocity $\varvec{u}$ and the hemodynamic indicators $\varvec{\theta }_{\text {hemo}}$ are integrated into the coupling framework. Within each coupling step $\Delta t_w$, we consider two approximation approaches, described below.

#### Steady formulation

Following the quasi-steady approach, the fluid domain is considered constant $\Omega _b = \Omega _b(\varvec{x}_b^{(n)})$ within $\Delta t_w$, since ISR-induced changes in the fluid are minimal between coupling steps. The steady-state blood velocity $\overline{\varvec{u}}$ is obtained by neglecting the time derivative in (2) and imposing a constant flow rate $\bar{Q} = \displaystyle \frac{1}{T_{HB}} \int _{\Delta T_{HB}} Q(t)~dt = 1.073~\text {[ml/s]}$, calculated over one cardiac cycle $\Delta T_{HB}$ (see Fig. 3b). Note that, in this formulation $\text{OSI}$ cannot be computed and that the definition of $\text{WSS}$ and $\text{TAWSS}$ coincide. We verify the soundness of this simplification in Fig. 3c. To distinguish the $\text{WSS}$ distribution obtained from the steady formulation, we refer to its tensor form and magnitude as:

4 $$\begin{aligned} \overline{\varvec{\tau }} =2 \mu _b (\overline{\varvec{E}}\varvec{n}_b - [(\overline{\varvec{E}}\varvec{n}_b)\cdot \varvec{n}_b]\varvec{n}_b), \qquad \text {and} \qquad \text {S-WSS}= \vert \overline{\varvec{\tau }} \vert, \end{aligned}$$

where $$\overline{\varvec{E}}:= \varvec{E}(\overline{\varvec{u}})$$.

#### Time-averaged formulation

To verify the steady approximation, we investigate whether deriving the $\text{WSS}$ distribution as time-averaged $\varvec{\tau }$ from a pulsatile flow field is comparable to $\overline{\varvec{\tau }}$. A transient solution over each coupling time step $\Delta t_w$ would be computationally expensive due to the small time step $\Delta t_b$ required for the fluid problems. However, we expect to observe a time-periodic flow field that follows the heartbeat. Hence, a simulation spanning a few cardiac cycles $\Delta T_{HB}$ over the total time $T_b$ allows the analysis of the characteristic flow field within every coupling step. The flow simulation is run from initial conditions until a periodic state is achieved, then all coupling variables are averaged over one heartbeat. Since the elastic response of the stented artery wall to blood pressure is in equilibrium and the lumen-wall interface $\Gamma _{b,w}$ moves only in a quasi-rigid manner, the formulation of (2) remains fully Eulerian. The hemodynamic indicators in (3) can be simplified, such that the integrals become $\displaystyle \frac{1}{T_b} \int _0^{T_b} \bullet \rightarrow \frac{1}{T_{HB}} \int _{\Delta T_{HB}} \bullet$, where $\Delta T_{HB}$ is a cardiac cycle in the periodic regime.

### Drug release

To model drug release, we account for two processes: (1) drug release into $\Omega _w$ by direct contact between stent and arterial wall on $\Gamma _{s,w}$ and (2) drug release into the bloodstream $\Omega _b$ through the luminal side of the stent $\Gamma _{s,b}$, followed by drug absorption from the blood into the arterial wall at the interface $\Gamma _{b,w}$. The drug concentrations in the lumen and arterial wall are denoted by $c_b$ and $c_w$, respectively. We adopt a quasi-steady formulation for the fluid and an unsteady formulation for the solid, as outlined in Algorithm 1(lines 7 and 8). For simplicity, drug dynamics within the polymer coating are assumed negligible, though depletion within the coating using a Higuchi-like model^71,72^ could be added in a later work.

For drug release in the arterial wall $\Omega _w$, the concentration $c_w$ is governed by the following unsteady advection-diffusion-reaction equation and boundary conditions:

5 $$\begin{aligned} {\mathscr {C}}_w(c_b): \qquad \begin{aligned}&\frac{\partial c_w}{\partial t} + \nabla \cdot (c_w {\varvec{u}}_w)-D_w \Delta c_w + \epsilon ~\rho _{{}_{\text {SMC}}}~c_w = 0 \quad && \text {in} \quad \Omega _w \times (0,T)\\&c_w = 0 \quad && \text {in} \quad \Omega _w \times \{ t= 0 \}\\&D_w \nabla c_w \cdot \varvec{n}_w = f_{B2}(t) \bar{q}^D_{w} \quad && \text {on} \quad \Gamma _{s,w}\\&D_w \nabla c_w \cdot \varvec{n}_w = P(c_b - c_w) {\mathscr {H}}\left( c_b - c_w\right) \quad && \text {on} \quad \Gamma _{b,w},\\&D_w \nabla c_w \cdot \varvec{n}_w = 0 \quad && \text {on} \quad \Gamma _w \setminus \left( \Gamma _{b,w} \cup \Gamma _{s,w} \right) \end{aligned} \end{aligned}$$

where $D_w = 0.05~[\text {mm}^2\text {/day}]$ is the diffusion coefficient, ${\varvec{u}}_w$ is the bulk wall velocity, and $\epsilon$ is the coefficient of receptor drug internalization on SMCs density $\rho _{{}_{\text {SMC}}}$ (see Eq. (9) for more details about the solid model setup). In the boundary conditions, $P = 0.1~\text {[mm/day]}$ is the endothelial permeability, ${\mathscr {H}}$ is the Heaviside function, $\bar{q}^D_{w}$ is the peak of the flux profile, and $f_{B2}(t)$ is a time-dependent factor describing the drug release rate from the stent, based on experimental values^63^. The time domain is $t \in (0, T)$, with $T = N \Delta t_w$.

The drug release into the blood stream is modeled by an unsteady advection-diffusion equation, if we consider the variation of concentration $c_b$ within the fluid time scale $T_b$:

6 $$\begin{aligned} \frac{\partial c_b}{\partial t} - D_b \Delta c_b + \varvec{u}(t) \cdot \nabla c_b = 0 \quad \text {in} \quad \Omega _b \times (0, T_{b}) \end{aligned}$$

where $D_b = 0.0001~[\text {mm}^2\text {/s}]$ is the diffusion coefficient. Similarly to the hemodynamics, the drug released into the bloodstream must be homogenized over the $\Delta t_w$. Below, we propose a time-averaging approach.

#### Steady and time-averaged formulations

In a quasi-steady regime, we assume instantaneous drug release, with concentration unchanged over $\Delta t_w$. For a generic time step $n$, where the beginning of the day is denoted as $t^{(n)}_{-}$ and the end as $t^{(n)}_{+}$, this is equivalent to setting $c_b\left(t^{(n)}_{-}\right) = c_b\left(t^{(n)}_{+}\right)$^1^.

Thus, the steady formulation becomes:

7 $$\begin{aligned} {\mathscr {C}}_b (\varvec{u}, \varvec{x}_b): \qquad \begin{aligned}&- D_b \Delta c_b + \overline{\varvec{u}} \cdot \nabla c_b = 0 \quad && \text {in} \quad \Omega _b (\varvec{x}_b)\\&c_b = 0 \quad && \text {on} \quad \Gamma _{\text {in}},\\&D_b \nabla c_b \cdot \varvec{n}_b = F_{B2}\bar{q}^D_{b} \quad && \text {on} \quad \Gamma _{s,b},\\&D_b \nabla c_b \cdot \varvec{n}_b = 0 \quad && \text {on} \quad \Gamma _{b,w} \cup \Gamma _{\text {out}}, \end{aligned} \end{aligned}$$

with $\bar{q}^D_{b}$ the peak of the flux profile, and $\displaystyle F_{B2} = F_{B2}^{(n)} = \frac{1}{\Delta t_w} \int _{t^{(n)}_{-}}^{t^{(n)}_{+}} f_{B2}(t)~dt \approx f_{B2}\left( {t}^{\left( n+\frac{1}{2}\right) }\right)$. The approximation of $f_{B2}(t)$ to $F_{B2}^{(n)}$ is justified in Fig. 3f.

#### Boundary conditions

We want to draw particular attention to the boundary conditions imposed on the stent $\Gamma _s$ and on the interface $\Gamma _{b,w}$.

To ensure consistency of drug released from $\Gamma _s$ between the fluid and solid models, we have to relate the fluxes on $\Gamma _{s,w}$ in (5) and $\Gamma _{s,b}$ in (7). Defining with $\bar{q}_D$ the peak of the flux profile from the whole stent $\Gamma _s$, the following conditions are imposed on the drug fluxes $\bar{q}^D_{b}$ and $\bar{q}^D_{w}$:

8 $$\begin{aligned} \bar{q}^D_{b,\text {[day]}} = C_{\text {sec}}^{\text {day}} \bar{q}^D_{b}, \qquad \bar{q}^D_{b,\text {[day]}} = \frac{1}{C^D_{bw}} \bar{q}_D, \qquad \bar{q}^D_{w} + \bar{q}^D_{b,\text {[day]}}= \bar{q}_D, \end{aligned}$$

where $C_{\text {sec}}^{\text {day}}=86400~\text {[s/day]}$ is the conversion factor of $\bar{q}^D_{b}$ from $\text {[fmol/mm/s]}$ to $\text {[fmol/mm/day]}$, $\bar{q}_D$ can be tuned according to the flux peak from the overall stent $\Gamma _s$, and $C^D_{bw}$ is a parameter used to tune the drug ratio between the abluminal side $\Gamma _{s,w}$ and the luminal side $\Gamma _{s,b}$.

All parameters for the fluid models are tuned to the time scale of seconds to reflect the dynamics of blood flow. Consequently, phenomena occurring at the lumen-wall interface can influence $c_b$ only if they operate on a comparable time scale. However, significant changes in $c_w$ occur over days, making standard Robin-Robin coupling conditions^66^ on $\Gamma _{b,w}$ infeasible in (5) and (7). Given the hydrophobic nature of the drug, we adopt Neumann-Robin boundary conditions on $\Gamma _{b,w}$, inspired by the concept of semi-permeable membranes^73^. On the fluid side, under the quasi-steady assumption, an equilibrium condition is imposed in (7) at the interface $\Gamma _{b,w}$, using homogeneous Neumann conditions. If no absorption occurs, the drug concentration $c_b$ is washed out by the blood flow^28,74^. Furthermore, lypophilic drugs are unlikely to be released back into the bloodstream once bound to the artery wall. Hence, we impose a *one-way* flux of drug absorption from the lumen into the wall. This is enforced in (5) using a Heaviside function to activate drug absorption at $\Gamma _{b,w}$ only when $c_b > c_w$; otherwise, a zero-flux condition is applied.

### Cell dynamics and growth model

The fluid-solid model proposed in this work extends a previously developed modeling framework for the solid-domain components^63^ – i.e., growth mechanics, arterial cell dynamics, and pharmacokinetics within the arterial wall (shown in green in Fig. 1a). Hence, in this section we outline only the main aspects of the solid model to provide the necessary context.

Concerning the cell dynamics (integrated in Algorithm 1, line 9), the interactions in the vessel wall between PDGF, TGF-$\beta$, ECM, SMCs, and ECs can be represented by a system of coupled advection-diffusion-reaction equations of the form

9 $$\begin{aligned} \Psi \left( c_w,\varvec{\theta }_{\text {hemo}}\right) : \qquad \begin{aligned}&\underset{{\text {rate}}}{\displaystyle {\left. \frac{\partial \phi }{\partial t}\right \vert _{\varvec{x}}}} + \underbrace{\nabla \cdot \left( \phi \,{\varvec{u}}_w\right) }_{{\text {advection}}} - \underbrace{\nabla \cdot \left( k_{\phi }\,\nabla \phi \right) }_{{\text {diffusion}}} - \underbrace{\overset{\text {source}}{{\mathscr {R}}(c_w)} + \overset{\text {sink}}{{\mathscr {S}}(c_w)}}_{{\text {reaction}}} = 0 \quad && \text {in} \quad \Omega _w \times (0,T),\\&\phi (\varvec{x}, 0) = \phi ^0 (\varvec{x}) \quad && \text {in} \quad \Omega _w \times \{ t = 0\},\\&- \varvec{q}_{\phi }\cdot \varvec{n}_w = \bar{q}_{\phi } \left( \varvec{\theta }_{\text {hemo}}\right) \quad && \text {on} \quad \Gamma _w, \end{aligned} \end{aligned}$$

for a scalar field $\phi$, where ${\varvec{u}}_w$ refers to the velocity of the vessel wall, $k_{\phi }$ is the diffusivity of the constituent $\phi$, $\bar{q}_{\phi }$ is the normal flux through the artery wall boundaries and initial conditions are set with $\phi ^0$. Here, we compactly present the cell species equations in Eulerian form to emphasize the individual contribution of each term. However, the processes in $\Omega _w$ – including the drug release in (5) and the cell dynamics in (9) – are solved in a Lagrangian framework and their detailed derivation is provided in a previous work^75^. All equations, except that for the EC density $\rho _{{}_\text {EC}}$, are solved in the bulk of the vessel wall. The endothelium, being lined with a monolayer of ECs, requires the EC density field to be modeled only on the luminal surface $\Gamma _{b,w}$. Hence, homogeneous Neumann boundary conditions are prescribed only at the boundary of the lumen-wall interface, $\partial \Gamma _{b,w}$. Initial conditions define denuded regions with zero density and healthy regions with a physiological equilibrium density of endothelial cells, $\rho _{{}_{\text {EC},eq}}$. Chemotaxis and haptotaxis of SMCs are accounted for by prescribing the effective advective velocity of SMCs as ${\varvec{u}}_w^{\text {eff}}:= \tilde{{\varvec{u}}}^{\text {eff}}({\varvec{u}}_w,c_{{}_P},c_{{}_C},\rho _{{}_\text {SMC}})$, where ${\varvec{u}}_w$ is the bulk wall velocity, $c_{{}_{P}}$ is the local PDGF concentration, $c_{{}_C}$ is the local collagen concentration in the ECM, and $\rho _{{}_\text {SMC}}$ is the local SMC density. For more insights on the coupling of cell dynamics and drug concentration $c_w$ in the artery wall, the interested reader is referred to earlier work^63^.

The effect of hemodynamic indicators on ISR growth is introduced through their influence on the increased concentration of PDGF, $c_{{}_P}$, and TGF-$\beta$, $c_{{}_T}$. In the absence of hemodynamic effects, the normal flux on $\Gamma _{b,w}$ is determined mainly by the EC density, $\rho _{{}_\text {EC}}$, scaled to its equilibrium value $\rho _{{}_{\text {EC},eq}}$. However, experimental evidence suggests that disturbed hemodynamics affect endothelial cell morphology. Even when the endothelial layer of the neointima is restored, cells under disturbed flow tend to adopt a rounder, less elongated shape compared to the healthy case^7^. This abnormal endothelial morphology promotes inflammation, platelet activation, and monocyte infiltration into the arterial wall, stimulating production of PDGF and TGF-$\beta$. To model these experimental observations, we introduce a shape index ($\text {SI}$) based on Saez et al.^76^, and incorporate the resulting enhanced PDGF and TGF-$\beta$ production through a flux condition at the lumen-wall interface. Below we summarize the Neumann conditions on $\Gamma _w$ for all cell species:

10 $$\begin{aligned} \begin{aligned}&\bar{q}_{{}_{P/T}}(\varvec{\theta }_{\text {hemo}}) = f_{B1}(t) \left[ 1- \left( \frac{\rho _{{}_{\text {EC}}}}{\rho _{{}_{\text {EC},eq}}} \right) \left( \frac{\text {SI}_{\min }}{\text {SI}(\varvec{\theta }_{\text {hemo}})}\right) \right] \bar{q}^{\text {ref}}_{{}_{P/T}} \quad && \text {on} \quad \Gamma _{b,w},\\&\bar{q}_{{}_{C/\text {SMC}}} = 0 \quad && \text {on} \quad \Gamma _{b,w},\\&\bar{q}_{{}_{\phi }} = 0 \quad && \text {on} \quad \Gamma _w \setminus \Gamma _{b,w}, \end{aligned} \end{aligned}$$

where $f_{B1}$ is a factor defining the flux profile in time^63^, and $\text {SI}(\varvec{\theta }_{\text {hemo}}) = \max \left\{ \text {SI}_{\min }, \exp \left[ -s_{{}_{\text {EC}}} \left( 1 - \text {OSI}\right) ^4 \text {WSS}\right] \right\}$. The shape index $\text {SI}(\varvec{\theta }_{\text {hemo}})$ enables the coupling mechanism with the hemodynamics, specifically affecting only PDGF and TGF-$\beta$. Here, $\text {SI}_{\min }$ is the asymptotic minimum shape index set to 0.05, and $s_{{}_{\text {EC}}} = 0.8$ is a model parameter. The SI curve and its parameters are derived to best fit the values in literature^76^.

The arterial wall structure is modeled using a compressible Holzapfel-Gasser-Ogden (HGO-C) hyperelastic formulation^77,78^, with parameters fitted for human coronary arteries^18^. A detailed discussion and evaluation of this constitutive model are provided in previous work^68^. The volumetric growth is consequently achieved via the kinematic prescription of the growth stretch tensor $\varvec{U}_g:= \tilde{\varvec{U}}_g(\tilde{\rho }_{{}_\text {SMC}})$ as a function of the local SMC density in the reference configuration $\tilde{\rho }_{{}_\text {SMC}}$. The quasi-static balance of linear momentum forms the basis for the structural problem, which is also solved in a Lagrangian framework^75^, and can compactly be represented as

11 $$\begin{aligned} \varvec{d}_{\text {ISR}} = {\mathscr {G}}(\varvec{U}_g(\phi )) \quad \text {in} \quad \Omega _w \times (0,T). \end{aligned}$$

The prediction of ISR displacement $\varvec{d}_{\text {ISR}}$ is integrated into the final step of the coupling scheme (Algorithm 1, line 10).

### Segmentation of the coronary OCT

**Figure 2:**
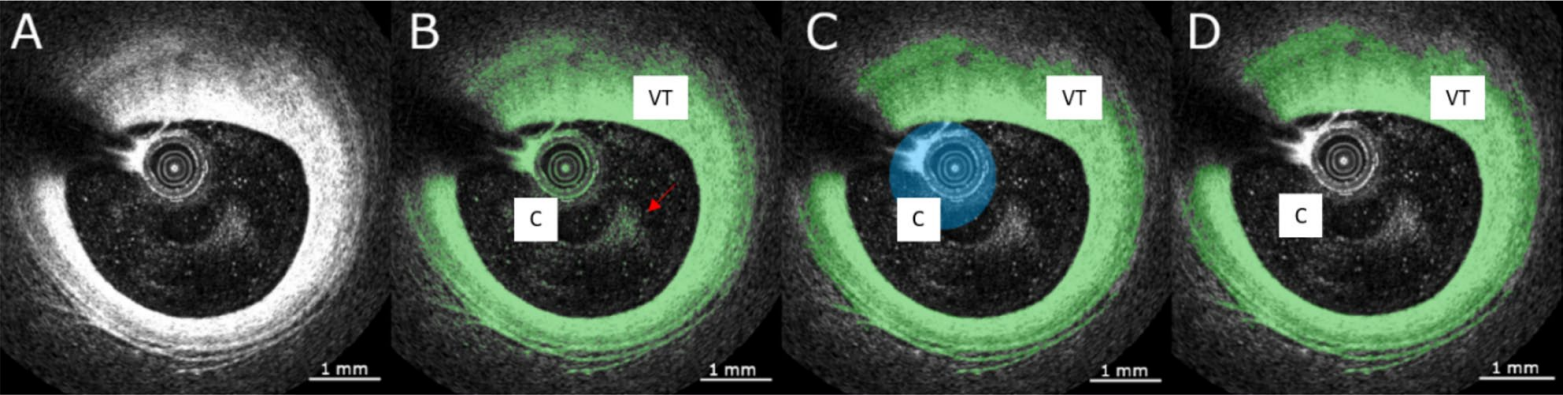
OCT imaging of the pre-stent intracoronary: (**A**) before segmentation; (**B**) after thresholding, vascular tissue, OCT catheter, and small particles within the lumen (red arrow) marked as tissue class (green); (**C**) segmentation of the OCT catheter (blue); (**D**) accurate segmentation of the vascular tissue (green). Legend: C = catheter, VT = vascular tissue.

A patient intracoronary OCT prior to the stent implantation was obtained by using ILUMIEN OPTIS-System . All data were anonymized and used for image segmentation in the Imalytics Preclinical v3.1.0.7 – Image analysis software by Gremse-IT (https://imalytics.com)^79^. All OCT images were set to Otsu Range as a standard threshold setting (Fig. 2A). Segmentation of the vascular tissue was performed, as showed in green, together with small artefacts in the lumen and the OCT catheter (Fig. 2B). The artefacts were removed and the OCT catheter was sequentially segmented along the vessel length (Sphere: $1-1.5~\text {[mm]}$ diameter, $1-2~\text {[mm]}$ length in section) and is displayed in blue (Fig. 2C). The remaining artefacts were segmented manually and small gaps within the segmented tissue were addressed using the morphological closing operation. This resulted in the display of segmented tissue without falsely marked artefacts, accurately representing the tissue area (Fig. 2D). For lumen segmentation, the vessel was outlined and the lumen was segmented using the Otsu thresholding method. Lumen artefacts were removed as previously described and the catheter segmentation was added to the lumen, resulting in the accurate segmentation of the lumen.

The intended retrospective study uses archived human data that is anonymised. No identifying information was extracted from the system and stored elsewhere. A statement by the RWTH Aachen University Hospital ethics committee (US EK 251-22) has been confirmed for the evaluation of coronary stents.

## Results

In this section, we analyze the mutual effects of hemodynamic indicators, drug transport and absorption, and ISR growth, after verifying the quasi-steady assumption. This study focuses on quantities related to interface and interaction phenomena, as those confined to the solid domain and blood flow modeling have been extensively analyzed in previous works^63,69,70^. We check the sensitivity of the main phenomena to one another using the simplified artery with a ring stent. We then present the main results of the coupled model on a more complex stented artery geometry derived from patient-specific OCT data and virtual stent implantation of a single-crown stent (see Fig. 1d). In both cases, the fluid mesh is unstructured with tetrahedral elements and boundary layers extending from $\Gamma _{b,w}$, whereas the solid mesh is structured with hexahedral elements. As shown in Fig. 1, these meshes are constructed so that the interface nodes match at $\Gamma _{b,w}$, eliminating projection errors and reducing computational cost during coupling. Since no processes occur within the stent itself, its volume $\Omega _s$ is not meshed. Moreover, we assume no stent malapposition in either case and no indentation^80^ for the simplified artery with a ring stent.

**Figure 3:**
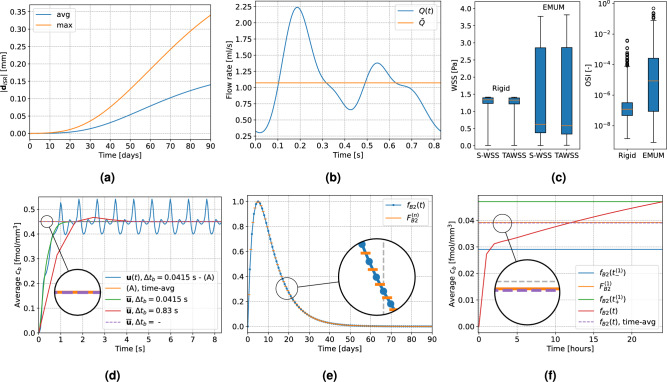
Quasi-steady approach verifiation on various quantities. (**a**) Average and maximum displacement from standalone simulation. (**b**) Blood flow rate on $\Gamma _{\text{in}}$. (**c**) Boxplots of $\text {S-WSS}$, $\text{TAWSS}$ and $\text{OSI}$, with rigid wall or deformation update via EMUM. (**d**) Evolution of average $c_b$ on $\Gamma _{b,w}$ in time, under different conditions. (**e**) Comparison of $F_{B2}^{(n)}$ and $f_{B2}(t)$. (**f**) Effect of $F_{B2}$ and $f_{B2}$ on $c_b$.

**Figure 4:**
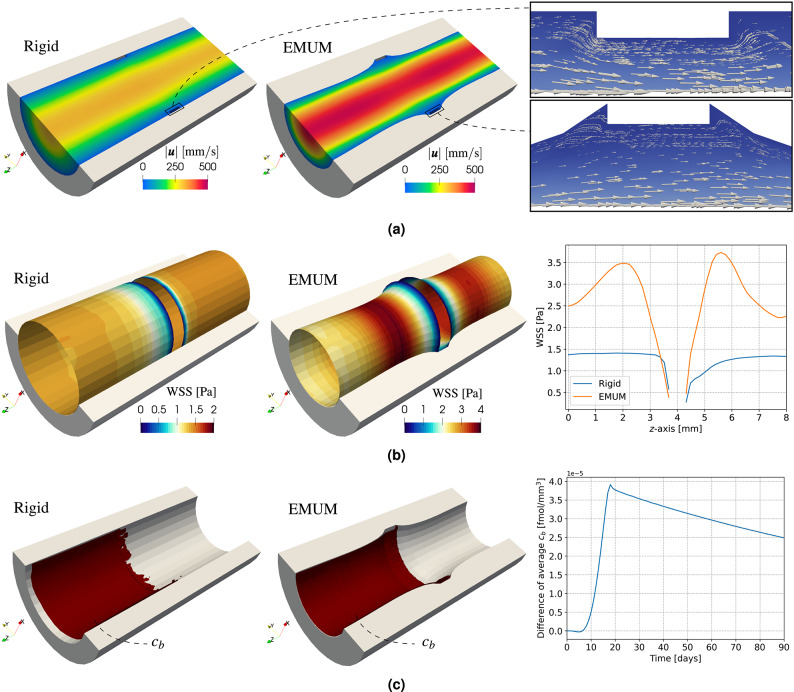
Effect of rigid wall assumption or deformation update via EMUM on hemodynamics and drug release, at fixed time $T = 90~\text{[days]}$. (**a**) Velocity magnitude and streamlines. (**b**) $\text {S-WSS}$ distribution and plot over line (right-most). (**c**) Isosurface of $c_b=10^{-4}~\text {[fmol/mm/s]}$ and average difference (right-most).

**Figure 5:**
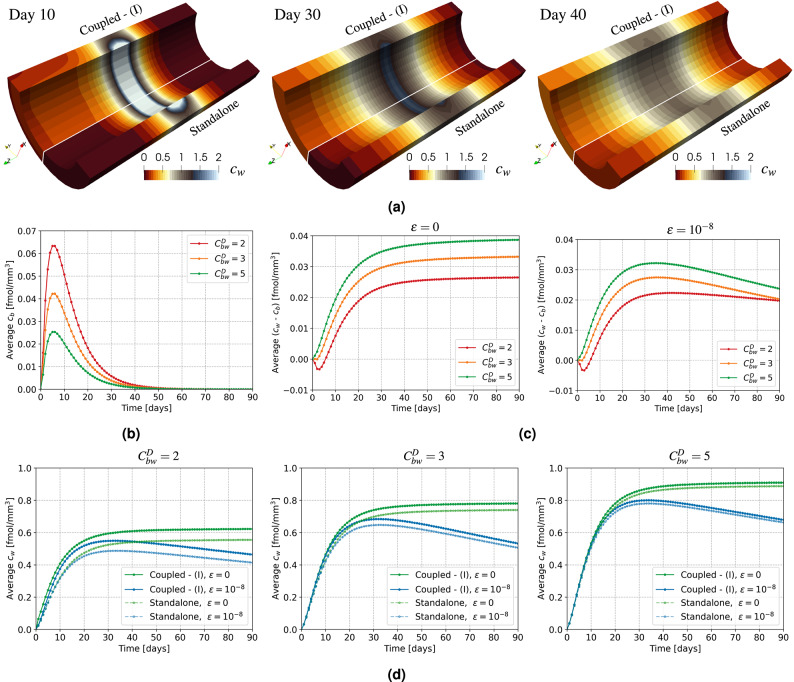
Evolution over $90$ days of $c_w$ and $c_b$ from standalone simulation or coupled case (I). (**a**) $c_w~[\text {fmol/mm}^3]$ at different time snapshots with $C^D_{bw} = 2$ and $\epsilon = 10^{-8}$. (**b**) Average $c_b$ over time for different $C^D_{bw}$. (**c**) Average $(c_w-c_b)$ on $\Gamma _{b,w}$ over time for different $C^D_{bw}$ and $\epsilon$. (**d**) Average $c_w$ over time for different $C^D_{bw}$ and $\epsilon$.

**Figure 6:**
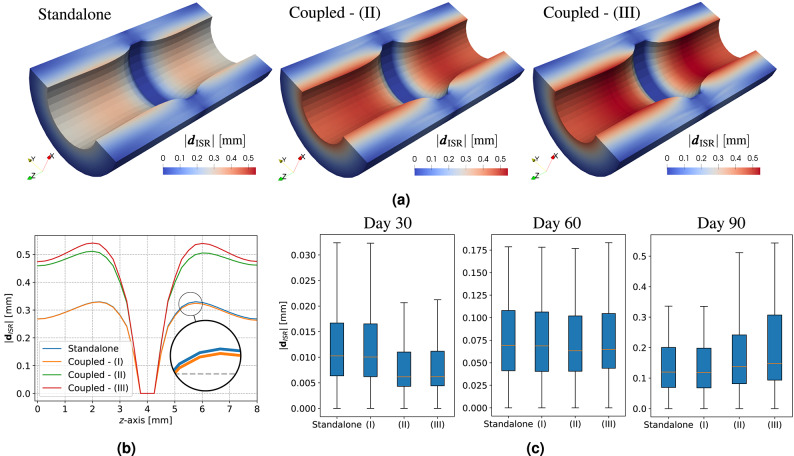
Effect of different coupling cases (I), (II) and (III) on wall displacement $\vert \varvec{d}_{\text {ISR}} \vert$. (**a**) Qualitative displacement after $90$ days. (**b**) Plot over line comparing displacement at day $90$. (**c**) Boxplots comparing displacement at different time snapshots.

In the next subsection, we evaluate the proposed model using a simplified test case with a ring stent by selectively activating or deactivating specific components and testing different configurations (e.g., steady vs. time-averaged formulations). An overview of all tests is provided in Table 1 – including figure references and labels used in the plots (if applicable) – where we indicate the presence, absence, or variation of each model component. In the last column (“Parameter variation”) we remark if specific component parameters are compared within the same configuration. This evaluation serves three main goals: (i) to assess whether the quasi-steady and quasi-rigid assumptions are acceptable (Fig. 3); (ii) to investigate whether certain interactions can be omitted without compromising model accuracy – specifically the impact of ISR displacement on hemodynamics and drug transport (Fig. 4), and the effect of coupling ${\mathscr {C}}_b$ on the drug concentration in the wall $c_w$ (Fig. 5); (iii) to identify which model or coupling component influences ISR progression the most (Fig. 6). Following the evaluation on the simplified setup, we apply the comprehensive coupling framework with all components to the patient-specific case.

**Table 1:**
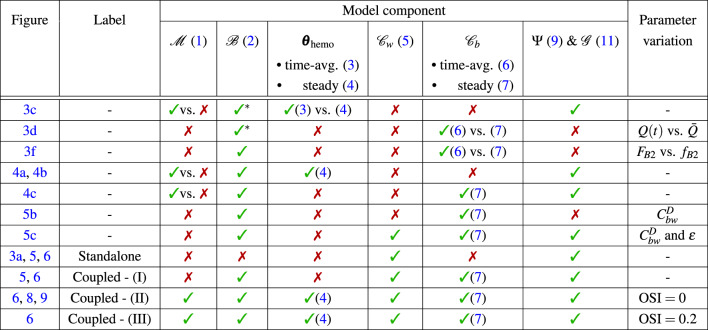
Overview of the test cases setup: model configuration and comparisons. The column “Figure” lists all figure references with the corresponding plot labels (if applicable) in the column “Label”. Figure references may appear multiple times if different coupling configurations are compared within the same plot. The columns under “Model component” list the components from Fig. 1a, their corresponding operators and time formulations (if applicable) with references to the Methods section. A  indicates that the corresponding component is active in the coupling framework, while a  indicates it is deactivated. The notation “vs.” denotes that two variants of the same component are compared within the test case. For ${\mathscr {M}}$,  corresponds to the *rigid* approach, and  to *EMUM*. The steady formulation of ${\mathscr {B}}$ is used by default, except for the first two rows (indicated by *), where the time-averaged (time-avg.) one is required by the corresponding variants of $\varvec{\theta }_\text {hemo}$ and ${\mathscr {C}}_b$. The flow rate profiles imposed on $\Gamma _{\text {in}}$ are shown in Fig. 3b, with *Q*(*t*) used for time-avg. and $\bar{Q}$ for steady. The column “Parameter variation” remarks which parameters are varied within a specific model component for comparison purposes. The curves for $F_{B2}$ and $f_{B2}$ are represented in Fig. 3e. The definitions of $C_{bw}^D$ and $\epsilon$ are given in (8) and (5), respectively.

For clarity, we use the following common labels among different tests (see also Table 1):

- Standalone: only the solid model is considered, with no influence from fluid or interface phenomena;
- Coupled - (I): includes the effects of drug release, specifically the contribution of $c_b$ to the solid model;
- Coupled - (II): incorporates hemodynamic effects into the solid model with $\text {OSI}= 0$;
- Coupled - (III): similar to Coupled - (II), but with $\text {OSI}= 0.2$.

Furthermore, for all tests, we use the following notations and abbreviations clarified here. The term *average* of a quantity refers to its spatial average within the corresponding domain $\Omega _*$. When averaging a quantity over time, we explicitly denote it as *time-avg*. Regarding wall deformation, we refer to a *rigid* wall when ISR deformation is not updated throughout the entire time horizon $(0,T)$. To describe the results from a quasi-rigid approach, we use the acronym *EMUM*, as we employ the elastic mesh update method to deform the lumen domain at each daily time step.

All single-field problems are discretized using the finite element method in space. For unsteady simulations, we employ the BDF2 multi-step method for the fluid domain^81^, while semi-implicit Backward-Euler method is used for the solid domain. The fluid problems are solved using the in-house solver XNS, and the solid problems are implemented into the commercial software FEAP v8.6 – A Finite Element Analysis Program (http://projects.ce.berkeley.edu/feap). All simulations are performed in parallel on the supercomputer CLAIX at RWTH Aachen University, using 96 cores for XNS and 48 or 96 threads for FEAP.

### Idealized artery with a ring stent

In this section, we present the main results for the test case, i.e., an idealized artery segment with a one-ring stent. The geometry includes lumen radius of $1.5~\text {[mm]}$; artery length of $8~\text {[mm]}$; wall thickness of $0.4~\text {[mm]}$ for the media layer and $0.3~\text {[mm]}$ for the adventitia; and ring stent with thickness of $0.1~\text {[mm]}$ and length of $0.5~\text {[mm]}$. The computational meshes consist of 8192 hexahedral elements for the solid and 135,672 tetrahedral elements for the fluid. Each daily time step requires about two minutes of computational time, running on 48 threads (FEAP) and 96 cores (XNS).

#### Verification of quasi-steady approach

In this section, we verify the assumptions made in the Methods concerning the suitability of the quasi-steady approach.

First, we test if the zero-order predictor for $\varvec{d}_{\text {ISR}}$, i.e., taking the displacement values from day $n-1$, is a satisfying approximation to update the lumen deformation at day $n$. As shown in Fig. 3a, the average displacement magnitude is approximately linear in time, ranging from $0$ to about $0.15~\text {[mm]}$ over $70~\text {[days]}$ (starting from day $20$, where the displacement becomes non-negligible). This corresponds to a daily increase of about $0.002~\text {[mm]}$, which is small enough to have a negligible effect on hemodynamic indicators. For completeness, the maximum displacement over time is also plotted, since some wall regions – especially those near the ring stent – are subject to more growth. In that case, the daily increase is about $0.004~\text {[mm]}$, which is still in the order of $10^{-3}~\text {[mm]}$.

Regarding blood flow modeling, inflow boundary conditions depend on the specific vessel. In our case, we focus on coronary arteries and base our inflow profile on experimental data for the right coronary artery^70,82^. The pulsatile profile $Q(t)$ is periodic in $(0, T_{HB})$, while the steady flow rate $\bar{Q} = 1.073~\text {[ml/s]}$ is set to the average of $Q(t)$ (see Fig. 3b). From the imposition of $Q(t)$ and $\bar{Q}$ inflow profiles, we compare the corresponding distributions of $\text{TAWSS}$ and $\text {S-WSS}$, at day $90$ after PCI (see Fig. 3c, left), for both rigid and quasi-rigid approaches. Each pair of boxplots with the same deformation update strategy reveals minimal differences in their median values and overall distributions. However, comparing rigid versus EMUM shows a greater impact on the $\text{WSS}$ values. Specifically, in the EMUM case, the median of $\text {S-WSS}$ decreases from a physiological $\approx 1.2~\text {[Pa]}$ in the rigid scenario to $\approx 0.6~\text {[Pa]}$, while the overall distribution becomes more spread out, with values below $0.5~\text {[Pa]}$ and above $1.5~\text {[Pa]}$. Further details about the spatial distribution of $\text{WSS}$ are provided in Fig. 4b.

Because $\text{WSS}$ can be reasonably approximated under steady conditions, we next examine $\text{OSI}$, whose computation relies on unsteady flow. To assess the impact of wall deformation on $\text{OSI}$, we compare results at day $90$ for both rigid and deformed lumen via EMUM. If the $\text{OSI}$ distributions are sufficiently similar, it is possible to run a single steady-state simulation to obtain $\text{WSS}$ and then assign a constant value for $\text{OSI}$ over time. Indeed, Fig. 3c (right) shows that although wall deformation does affect the $\text{OSI}$ distribution, its median changes only from about $10^{-7}$ to $10^{-5}$. $\text{OSI}$ values in the order of $10^{-1}$ occur only in the EMUM case and are sparsely distributed, confined mostly to regions near the stent. To reduce computational effort, we test the impact of different $\text{OSI}$ values, by setting it to a constant value on the whole interface.

The choice of a pulsatile or a steady flow rate also affects the drug distribution. Assuming arbitrary values of $F_{B2} = 1$ and $\bar{q}^D_{b} = 0.5~\text {[fmol/mm/s]}$ for testing purposes, we verify the quasi-steady approach on the drug released from the stent into the blood stream. In Fig. 3d we compare the spatially averaged concentration $c_b$ at the interface $\Gamma _{b,w}$ obtained from (7) with the transient solution of (6) over $10$ heartbeats. We also test the results from solving (6) with a steady advection field $\overline{\varvec{u}}$. Regardless of the choice of $\Delta t_b$, all transient solutions coupled to $\overline{\varvec{u}}$ reach the same steady state within a few time steps. The unsteady solution oscillates around this steady state, with peaks at the highest and lowest flow rates. The time-averaged concentration is slightly higher than the steady-state value, but the difference is on the order of $10^{-4}$. Because the effect of $c_b$ on the drug absorption in (5) is further scaled by $P$ on $\Gamma _{b,w}$, we choose to use (7) to compute $c_b$, thereby reducing computational cost without significantly compromising accuracy.

Another key assumption is that the flux $F_{B2}\bar{q}^D_{b}$ from $\Gamma _{s,b}$ is instantaneous and remains constant over each day. However, especially in the early days, the function $f_{B2}(t)$ can exhibit considerable daily jumps (see Fig. 3e). For example, $f_{B2}(t_{-}^{1}) = f_{B2}(1~\text {[day]}) \approx 0.2284$ and $f_{B2}(t_{+}^{1}) = f_{B2}(2~\text {[days]}) \approx 0.3698$. To evaluate whether $F_{B2}^{(1)}$ is a suitable approximation between the beginning and end of day $1$, we compare three steady solutions of (7) with constant drug release (using the following values, separately: $F_{B2}^{(1)}$, $f_{B2}(t_{-}^{1})$, and $f_{B2}(t_{+}^{1})$) to the solution of (6) with $\varvec{u}(t) = \overline{\varvec{u}}$, $\Delta t_b = 1~\text {[hour]}$, and a time-varying drug release $f_{B2}(1 + t/24)$ over $24$ hours. The choice of $\Delta t_b = 1~\text {[hour]}$ is justified by the linearized hourly variation of $f_{B2}$, which remains small, i.e., $\frac{0.3698 - 0.2284}{24} \approx 0.005$. We focus on the interval from day $1$ to day $2$, because this is where one of the largest daily changes occurs. Thus, if the approximation of $f_{B2}$ with $F_{B2}$ is valid on day 1, then we can safely assume that it is valid for all remaining days. Among the three steady drug distributions in Fig. 3f, the difference in average $c_b$ is in the order of $10^{-2}$. Notably, the midpoint strategy $F_{B2}^{(1)}$ best approximates the time-averaged $c_b$ resulting from the imposition of unsteady hourly drug release. This indicates that the assumption on $F_{B2}$ is sufficiently accurate for practical purposes.

#### Influence of growth on hemodynamics

In this section, we assess whether updating the lumen deformation via EMUM (see (1)) affects hemodynamic quantities and the drug release into the bloodstream. Based on the previous verification (see Fig. 3), all results presented here are derived from the quasi-steady formulations of (2), (4) (i.e., $\text {WSS}= \text {S-WSS}$), and (7). We focus mostly on the final time $t=T$, set to $90$ days after PCI, since the maximum displacement occurs at this stage. If no influence is observed at $T$, we can reasonably conclude that earlier daily time steps are also unaffected.

We begin by examining the effect of deformation on velocity and streamlines. As shown in Fig. 4a, the velocity magnitude is substantially altered by deformation, increasing from about $250~\text {[mm/s]}$ to $500~\text {[mm/s]}$ and affecting boundary layers near the artery wall. In the rigid-wall scenario, the boundary layer is thicker, confining flow disturbance and recirculation mainly to the stent corners. Beyond these regions, including the length of the ring stent, the flow remains laminar. When deformation is introduced with EMUM, the boundary layer is compressed, producing steeper gradients near the wall and extending flow disturbance to the entire area around the stent rather than just its corners.

The altered velocity gradient and thinner boundary layer described above suggest a non-negligible impact on $\text{WSS}$. This is confirmed in Fig. 4b. Under the rigid-wall assumption, deviations from the physiological $\text{WSS}$ for coronary arteries (approximately $0.8~\text {[Pa]}$ to $1.5~\text {[Pa]}$) occur primarily near the stent, where recirculation zones and larger downstream vortices appear. As a result, a wider region with $\text{WSS}$ values up to $0.4~\text {[Pa]}$ is observed downstream of the stent. Introducing deformation to the blood domain (labeled as EMUM) increases the area of critically low $\text{WSS}$ both upstream and downstream of the stent. It also introduces higher non-physiological values above $2~\text {[Pa]}$, as seen more clearly in the line plot (Fig. 4b, right). Here, the steeper velocity gradient in the EMUM case generates both overshoot and undershoot in the $\text{WSS}$ distribution. By contrast, the rigid scenario maintains nearly physiological $\text{WSS}$ throughout most of the wall, except near the stent. Notably, in EMUM, the $\text{WSS}$ undershoots more on the upstream side of the stent than in the rigid case, whereas at $z=4.25~\text {[mm]}$ downstream of the stent corner, it becomes slightly higher, leading to a more pronounced overshoot further downstream.

The final aspect we investigate is whether deformation affects the drug distribution $c_b$. In Fig. 4c, we show isosurfaces of $c_b = 10^{-4}$ at $90$ days after PCI for both the rigid-wall and EMUM cases (left and center). In the EMUM scenario, likely due to increased recirculation, the isosurface is smoother, with slightly more drug accumulating near the stent and at the outflow. However, when examining the average $c_b$ over the entire timeline $(0,90)~\text {[days]}$, the difference is on the order of $10^{-5}$ (see Fig. 4c, right). This effect is negligible compared to the significant variations observed in the hemodynamics.

#### Coupled pharmacokinetics

In this section, we examine how coupling $c_b$ to drug absorption in the artery wall influences the overall drug distribution of $c_w$. Specifically, we set $\bar{q}_D = 1~\text {[fmol/mm/day]}$ and investigate the impact of receptor drug internalization on SMCs by comparing $\epsilon = \{ 0, 10^{-8} \}$, and different drug ratios $C^D_{bw} = \{2,3,5\}$, as defined in (8). Modern drug-eluting stents are designed to load more drug on the abluminal side to minimize washout by blood flow. However, precise data regarding the drug ratios between the luminal and abluminal sides are not available. Hence, we assume the drug flux from the luminal side is at most equal to that on the abluminal side, corresponding to $C^D_{bw} = 2$. The settings $C^D_{bw} = \{3,5\}$ indicate that the drug load on $\Gamma _{s,b}$ (luminal side) is one-third or one-fifth of the total drug load on $\Gamma _s$, respectively. For the computation of the drug released in the bloodstream $c_b$, we neglect lumen deformation based on the findings in Fig. 4c.

We begin by setting $\epsilon = 10^{-8}$ and $C^D_{bw} = 2$, which in our setup means the highest possible luminal drug flux among the three tested configurations. This setup enables us to observe how drug absorption affects the distribution of $c_w$ in the artery wall. In Fig. 5a, we compare the standalone solution for $c_w$ with the result obtained by coupling $c_b$. In the standalone case, drug remains symmetrically distributed around the ring stent at all times, even considering internalization. However, once $c_b$ is incorporated, blood advects the drug downstream, leading to asymmetric accumulation of $c_w$ in the artery wall and aligning more closely with experimental observations. This asymmetry is particularly evident at day $10$, when the drug from the stent has not yet diffused significantly into the artery wall, whereas advection quickly transports part of the drug downstream, where it is absorbed. As the drug supply in the stent depletes over time, more drug is diffused into the wall, reducing the concentration gradient between $c_b$ and $c_w$ and, thus, the amount of absorbed drug governed by the boundary condition on $\Gamma _{b,w}$ in eq. (5). Consequently, the effect of coupling $c_b$ diminishes in time (see for example snapshot at day $40$, right-most plot in Fig. 5a).

After identifying the regions where $c_b$ is absorbed, we notice that the drug in the bloodstream is substantially lower than the total amount in the artery wall, due to the highly advective nature of blood flow. Fig. 5b shows the average drug $c_b$ in the lumen $\Omega _b$ for different values of $C^D_{bw}$. As described earlier, $C^D_{bw} = 2$ exhibits the highest peak of $c_b$ because the drug flux is divided equally between the luminal and abluminal sides of the stent. In contrast, $C^D_{bw} = 3$ and $C^D_{bw} = 5$, involve higher fluxes on the abluminal side, allowing more direct diffusion into the artery wall. Moreover, the average $c_b$ remains on the order of $10^{-2}$, which aligns with the fact that the majority of the drug in contact with blood is quickly advected and washed out, while only a small portion near the boundary layer is absorbed into the artery wall. Hence, this value offers an approximate measure of the drug washed out from the stent into the bloodstream.

To determine when the one-way flux condition in (5) is activated, we compare $c_b$ and $c_w$ at the interface. In Fig. 5c, we plot the average difference $c_w-c_b$ over time and observe that this difference is negative mainly for $C^D_{bw}=2$ during the first $10$ days after PCI. Although this does not exclude drug absorption for other $C^D_{bw}$ values or at different time points, it suggests that negative $c_w-c_b$ occurs only in limited interface regions. Furthermore, the contribution of $c_b$ becomes less significant both at later stages after PCI, and for smaller drug loads on the luminal side. The choice of $\epsilon$, which determines whether drug internalization is considered, does not strongly influence early $c_b$ absorption because SMCs density increases gradually. Its effects on $c_w$ appear around day $20$, by which time the overall contribution of $c_b$ is already reduced compared to the initial $10$ days.

To summarize the effects of $c_b$ absorption, drug ratio $C^D_{bw}$, and drug internalization $\epsilon$, we present the time evolution of the average concentration $c_w$ for all parameter combinations in Fig. 5d. The main findings are:

- The average drug in the wall $c_w$ increases for higher $C^D_{bw}$, i.e., when more drug is loaded on the abluminal side of the stent $\Gamma _{s,w}$.
- The strongest influence of $c_b$ absorption occurs when setting $C^D_{bw}=2$.
- The difference in average $c_w$ between the standalone and coupled simulations remains approximately constant after day $20$, indicating that the effect of coupling $c_b$ primarily manifests during the first $10$ – $20$ days after PCI.
- For $\epsilon = 0$, the jump in average $c_w$ between standalone and coupled case is higher because no drug is internalized.

#### Effects of fluid and drug coupling on growth

In this section, we examine how coupling hemodynamics and drug absorption affects ISR growth, focusing on neointimal thickness, quantified as the wall displacement magnitude $\vert \varvec{d}_{\text {ISR}} \vert$. Based on the findings of the previous sections, we set certain parameters: all results presented here assume $C^D_{bw}=2$ and $\epsilon = 10^{-8}$ for the computation of $c_b$ and $c_w$; $\text{WSS}$ values are computed after lumen deformation via EMUM and quasi-steady approach, i.e., corresponding to $\text {S-WSS}$ in (4); the $\text{OSI}$ is held constant across the interface, tested once at $\text {OSI}=0$ (corresponding to “Coupled - (II)”) and once at $\text {OSI}= 0.2$ (marked as “Coupled - (III)”).

We begin with a qualitative assessment of wall displacement at $90$ days after PCI. Fig. 6a compares the standalone case with coupled cases (II) and (III). Including hemodynamic indicators produces a noticeable effect on neointimal thickness, showing about $0.2\text{[mm]}$ of additional displacement compared to the standalone scenario. We omit a qualitative plot of the coupled case (I) at this scale, as it exhibits no clear differences from the standalone case.

To further illustrate these effects, Fig. 6b shows the displacement magnitude along a line on the interface. Two main observations emerge. Firstly, although the difference between standalone and case (I) is minor, it highlights an essential characteristic of ISR, namely its asymmetry around the stent. In the standalone case, neointima growth is symmetric relative to the stent position – similar to the symmetric drug distribution $c_w$ in Fig. 5a. Adding $c_b$ absorption slightly reduces displacement on the downstream side, where higher drug uptake slows SMCs proliferation, leading to asymmetrical neointimal thickness. Secondly, the introduction of hemodynamic indicators exerts a much stronger influence on ISR displacement, confirming the qualitative findings in Fig. 6a. According to our implementation of $\text{OSI}$ in (10), setting $\text {OSI}=0.2$ further increases the thickness of approximately $0.01\text{[mm]}$. However, $\text{OSI}$ values of $0.2\text{[mm]}$ occur only near the stent, so accurately computing $\text{OSI}$ everywhere (via unsteady simulations) would offer limited improvements in predicting $\varvec{d}_{\text {ISR}}$. Because drug also plays a role in the coupled cases (II) and (III) the downstream asymmetry of wall displacement persists.

An additional observation is that the increased displacement $\varvec{d}_{\text {ISR}}$ seen in coupled cases (II) and (III) does not manifest during the first $30$ days after PCI. Fig. 6c illustrates the evolution of neointimal thickness at three time snapshots using boxplots, which show both median values and their ranges on the solid domain $\Omega _w$. During the initial $30$ days, EC density $\rho _{_{\text {EC}}}$ drives the flux term of PDGF and TGF-$\beta$ in (10), and hemodynamic changes remain limited. This leads to lower predicted displacement for the coupled cases at early times. As time advances, the EC layer is progressively restored, reducing PDGF and TGF-$\beta$ concentration in the standalone model. By day $90$, the EC layer is theoretically fully re-established, blocking the production of PDGF and TGF-$\beta$ in the standalone formulation. In contrast, altered hemodynamics in the coupled cases allow PDGF and TGF-$\beta$ fluxes to increase, even when EC density approaches its equilibrium level $\rho _{_{\text {EC}, eq}}$. The drug coupling (case (I)) does not substantially affect the overall thickness over time, but does influence its symmetry, as noted above.

### Towards patient-specific stented artery

In this final example we showcase the ability of the proposed methods to handle patient specific geometries. For this example we perform a virtual stent implantation, i.e., we take an artery segment from a patient OCT (see Fig. 2) and simulate the placement of the stent inside this artery. This stent placement process is modelled as a purely structural problem, neglecting any fluid effects. The deformed artery and stent geometries resulting from the virtual stent expansion are then converted to a suitable geometry for the fluid-solid simulation. In the next sections, we give a detailed overview over the individual steps taken for this example and then present the main results of the fluid-solid model.

#### Virtual stent implantation

**Figure 7:**
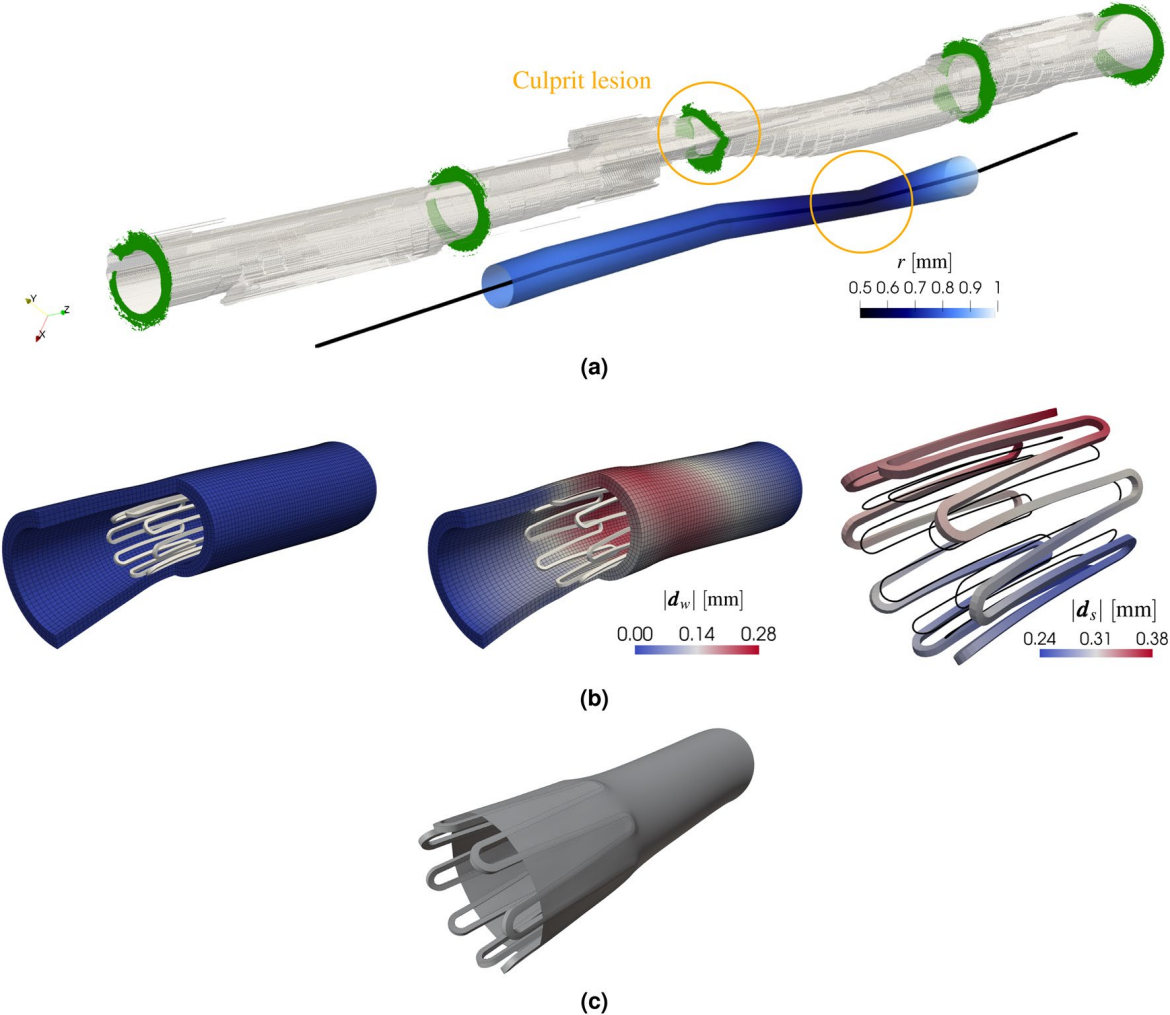
From OCT imaging to virtual stent implantation. (**a**) 3D reconstruction of lumen and 2D slices of artery wall from OCT data (top). Reconstruction of centerline and artery radii *r* (bottom). The culprit lesion is highlighted with a yellow circle. (**b**) Initial (left) and final (center) configuration of the virtual stent expansion. The right image shows a close-up of the deformed stent, where the initial stent centerline geometry is indicated with the black curve. The stent is modeled with 1D structural beam elements and is visualized with rectangular cross-section. The color bars show the norm of the structural displacements of the artery wall, $\varvec{d}_w$, and of the stent crown, $\varvec{d}_s$, respectively. (**c**) Post-processing of the artery and stent geometry, where the full 3D stent geometry is intersected with the deformed inner surface of the artery.

The reconstructed 3D lumen from the OCT data is $72\text{[mm]}$ long, with a diameter ranging from $3.042\text{[mm]}$ in the distal part of the artery to $1.363\text{[mm]}$ near the culprit lesion (see Fig. 7a, top). Using the open-source software Blender v4.5.2 (https://www.blender.org)^83^, we extracted the centerline and corresponding radii of the artery segment proximal and distal to the culprit lesion, as shown in Fig. 7a (bottom). Starting from this smooth artery segment, we simulated the stent expansion within the artery.

We employ an approach recently introduced by Datz et al.^52^. The main idea is to use standard three-dimensional (3D) structural finite elements for the artery and one-dimensional (1D) structural beam theories to model stent struts. This allows for a relatively simple creation of complex stent models (compared to full 3D stent discretizations) and also drastically reduces the number of degrees of freedom required to accurately describe the stent. The unilateral contact between the 1D stent and the 3D artery requires a mixed-dimensional contact scheme. The study of mixed-dimensional interactions has been an active area of research in recent years and we use a contact formulation which heavily builds upon the previous works^56–58^. We employ geometrically exact Simo-Reissner beam finite elements to model the struts^53^, which results in a highly accurate and efficient model for the stent structure.

The considered stent geometry is a single-crown prototype stent with 8 struts, which resembles the structure of a Resolute Integrity stent. The stent is $3~\text {[mm]}$ long, has an initial diameter of $0.9~\text {[mm]}$ and is placed at the position where the inner artery diameter is the narrowest, in medical terms the *culprit lesion*, see Fig. 7b (left). The stent strut has a square cross-section with a thickness of $0.1~\text {[mm]}$ and the stent material is assumed to be elastic with a Young’s modulus of $2.4\cdot 10^5~[\text {N/mm}^2]$ and a Poisson’s ratio of $0.3$. The artery wall is modeled as a hyperelastic, anisotropic material using a neo-Hookean strain energy function for the base material and an exponential strain energy function for the embedded collagen fibers^77^. In other words, the arterial wall is modeled with an HGO-C hyperelastic formulation, but with parameters fitted specifically for human coronary arteries^18^: for the media layer, with an assumed thickness of $0.4~\text {[mm]}$, the shear modulus for the matrix is set to $0.06~\text {[MPa]}$ while for the adventitia, it is set to $0.024~\text {[MPa]}$. The near incompressibility is accounted for through the prescription of very high bulk moduli for both the layers^63^. The collagen fibers are arranged in a double helix structure, i.e., two fiber families in opposite directions with a helix angle of $41$° for the media and $50.1$° for the adventitia. The parameters for the exponential strain energy function are $0.112~\text {[MPa]}$ and $0.362~\text {[MPa]}$; the exponential coefficients are $20.61$ and $7.089$ for the media and adventitia, respectively^18^. Dirichlet boundary conditions are applied on the stent such that all rigid body modes are constrained and that the stent can expand unobstructed in radial direction, while the artery is fixed at the beginning and at the end. The virtual stent implantation model is set up using the open source beam finite element pre-processor BeamMe^84^ and is simulated with the open source parallel multiphysics research code 4C^85^. The stent struts are loaded with a distributed line load of $0.01~\text {[N/m]}$, pointing outward in radial direction, which is applied in a linear manner over $100$ quasi-static time steps. The deformed configuration of the stented artery is shown in Fig. 7b (center). Moreover, Fig. 7b (right) shows a close up of the deformed stent. It can clearly be seen that the expansion of the stent expands the artery and that the artery is subject to large deformations. This deformed model, obtained by a purely structural mechanics simulation shall now be used to obtain suitable initial meshes for the fluid-solid simulation. We want to highlight, that the physical correctness of the employed parameters and material models is not the primary interest of this numerical example. We want to illustrate the ability of the proposed fluid-solid model to handle general non-trivial initial stent and artery geometries. For a more elaborate discussion of physically meaningful parameters for different arteries and stent properties in the mixed-dimensional interaction model, the interested reader is referred to Datz et al.^52^.

#### Generation of the initial stent artery geometry

To obtain the initial meshes for the fluid-solid simulation we have to post-process the simulation result obtained in the previous section. First, the stent cross section is extruded along the 1D beam centerline of the stent struts to obtain the full 3D geometry of the stent. This is visualized in Fig. 7b (left and center). In a next step, this 3D stent geometry is intersected with the inner (deformed) artery surface, see Fig. 7c. Due to the non-matching meshes in the contact simulation, we need to slightly extend the cross section geometry in radial direction to obtain unique solutions for the intersections. Now two volumes can be defined, i.e., the lumen volume, and the artery volume. The lumen volume is enclosed by the inner most stent surface, and the inflow and outflow surfaces of the considered blood vessel. In a similar manner, the artery volume is defined between the inner and outer artery surfaces. However, it is not required to model the artery over the full length of the considered geometry. This is justified because during stent implantation, we assume that the injured artery portion is closest to the stent. Furthermore, we use this case merely as proof-of-concept, not as accurate representation of a patient-specific scenario. Finally, the lumen and artery volumes are meshed to obtain a matching interface between them, see Fig. 1d.

#### Evaluation of coupled model

The results shown in this section were obtained using 241,668 hexahedral elements for the solid mesh, 652,309 tetrahedral elements for the fluid mesh, and approximately 10,000 interface nodes. Each daily time step required about 60 minutes of computation, employing 96 threads for FEAP and 96 cores for XNS. We analyze the main results of the fully coupled model using coupled setup (II), based on the observations from the test case. Furthermore, we highlight only the principal findings that differ notably from those already presented for the ring stent setup.

**Figure 8:**
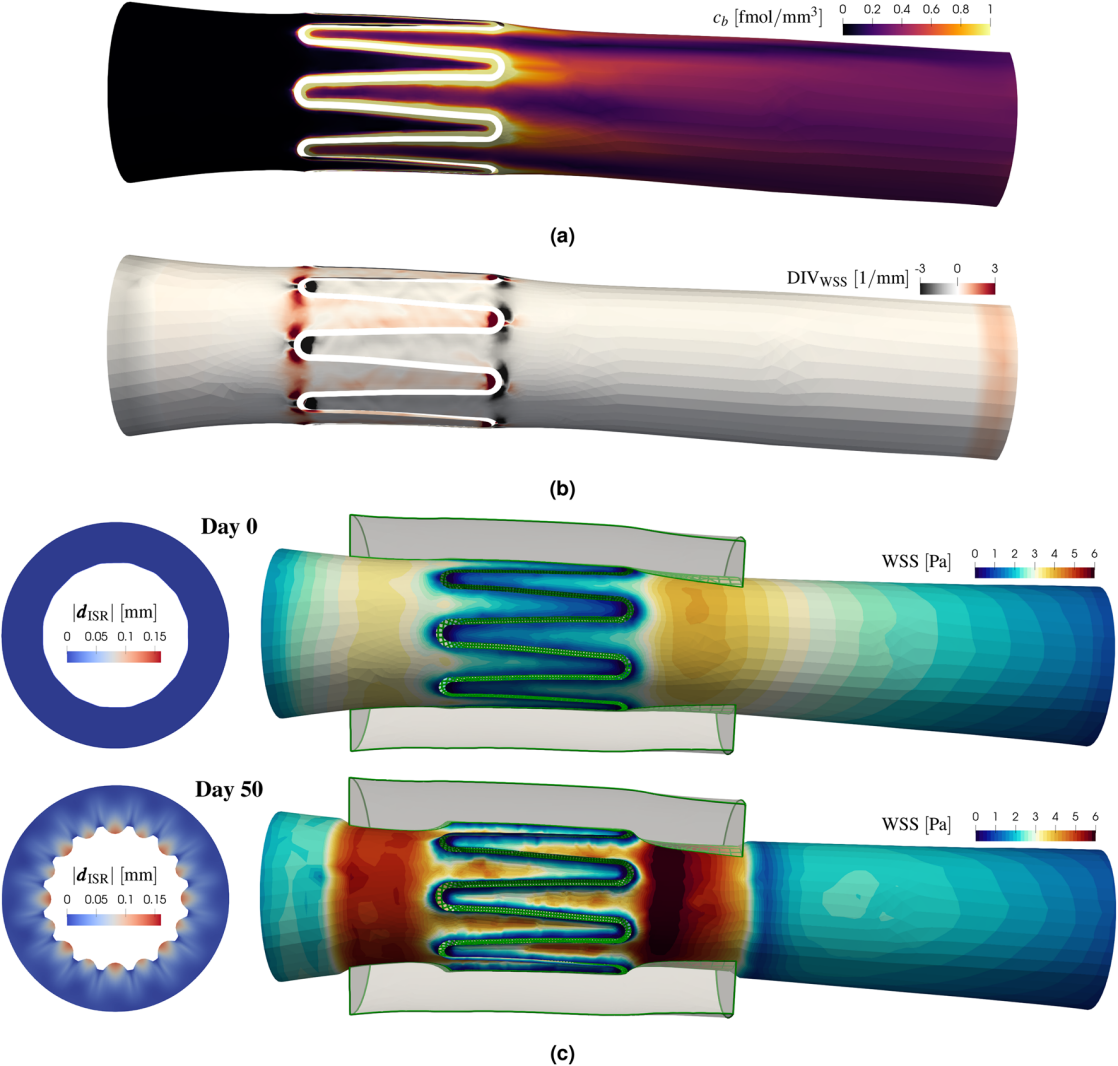
Results on patient-specific artery with single-crown stent. (**a**) Drug concentration $c_b$ at lumen-wall interface $\Gamma _{b,w}$. (**b**) Distribution of ${\text {DIV}}_{\text {WSS}}$. (**c**) Evolution of $\vert \varvec{d}_{\text {ISR}} \vert$ (on one radial slice) and $\text{WSS}$ distribution.

In particular, we consider the drug distribution $c_b$ on the lumen-wall interface $\Gamma _{b,w}$, as shown in Fig. 8a. Unlike the ring stent case, the drug preferentially accumulates along the curved portions of the stent, forming a distinct pattern downstream of certain struts. These regions coincide with negative values of the divergence of normalized $\text {WSS}$, defined as

$$\begin{aligned} {\text {DIV}}_{\text {WSS}}= \nabla \cdot \left( \frac{\overline{\varvec{\tau }}}{ \vert \vert \overline{\varvec{\tau }} \vert \vert _2} \right) . \end{aligned}$$

The divergence of the wall shear stress tensor ${\text {DIV}}_{\text {WSS}}$, characterizes regions of $\text{WSS}$ streamlines accumulation (negative values) versus expansion (positive values). Although the mesh resolution may not fully capture all local ${\text {DIV}}_{\text {WSS}}$ features, the results align well with previous studies^70^ and are sufficient for a qualitative assessment. As shown in Fig. 8b, the highest $c_b$ accumulation corresponds to areas of negative ${\text {DIV}}_{\text {WSS}}$, in agreement with in-silico observations on lipoprotein polarization^86^.

Another noteworthy result involves the interplay between neointimal thickness and $\text{WSS}$. Fig. 8c compares conditions at day 0 and day 50 after PCI. Specifically, it illustrates ISR growth on a radial slice through the artery midpoint and the $\text{WSS}$ distribution in a longitudinal view of the lumen. At day $0$, aside from minor deformation due to the virtual stent implantation, the hemodynamics already deviate from the idealized artery segment. Low $\text{WSS}$ arises near the stent due to recirculation, while the natural curvature of the coronary artery, especially downstream of the stent, produces locally elevated $\text{WSS}$ beyond physiological levels. By day $50$, neointima thickness reaches about $0.16~\text {[mm]}$, and the corresponding deformation significantly alters $\text{WSS}$, resulting in peaks up to $6~\text {[Pa]}$ downstream of the stent and deeper recirculation zones around the struts.

**Figure 9:**
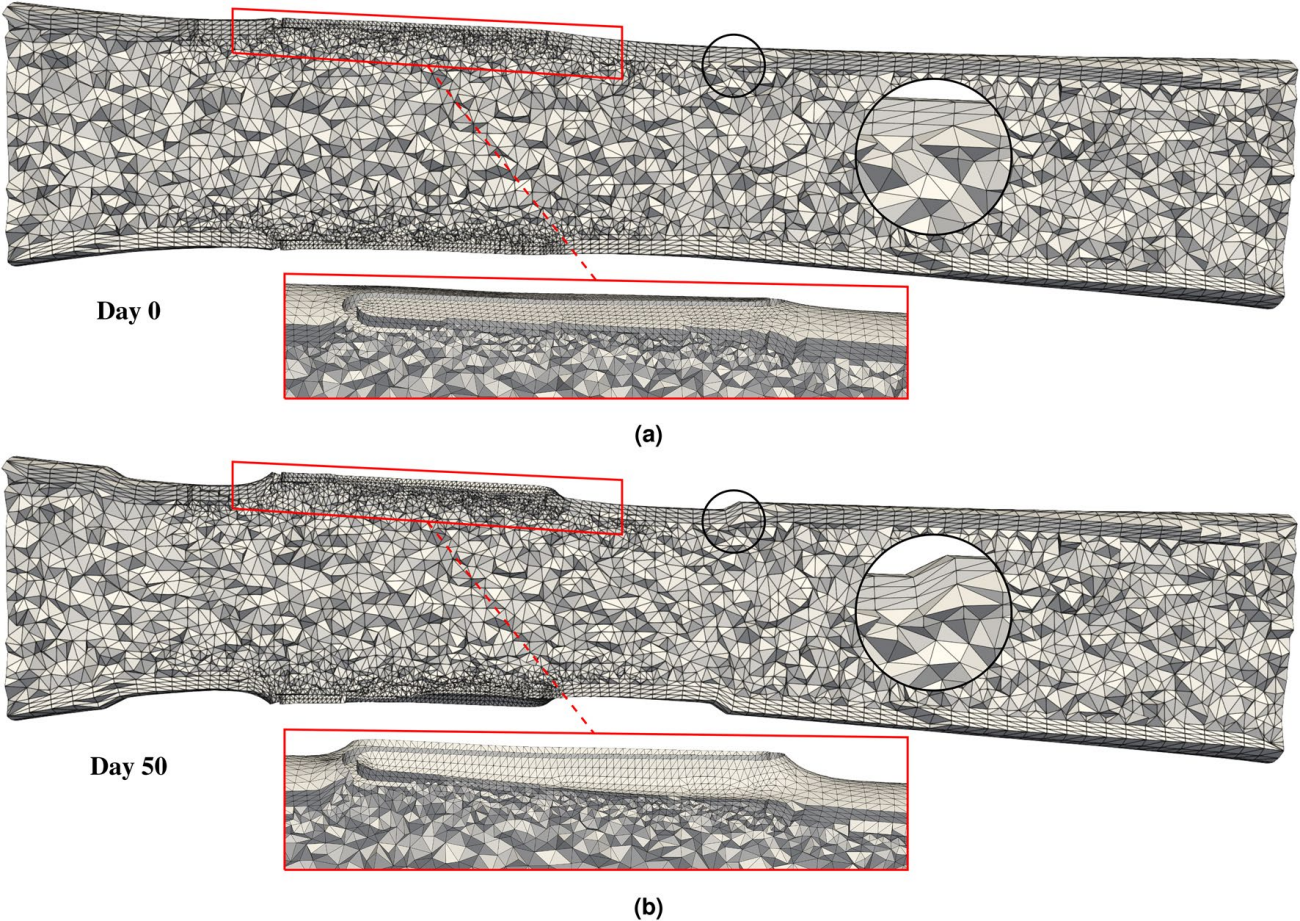
Longitudinal slice of the fluid mesh, highlighting boundary layers (black circle) and the refined region around the stent (red rectangle). (**a**) Mesh elements after virtual stent implantation, before ISR simulation. (**b**) Mesh elements after 50 days, including ISR deformation via EMUM.

In Fig. 9, we show how the fluid mesh is affected by the lumen deformation due to ISR. After virtual stent implantation, the mesh is generated with five boundary layers along the arterial wall and a refined unstructured tetrahedral region around the stent (Fig. 9a). This setup improves the resolution of near-wall phenomena, which is essential for capturing hemodynamic indicators and drug transport accurately. At day $50$, the predicted ISR growth $\varvec{d}_\text {ISR}$ is imposed at the interface $\Gamma _{b,w}$ and the mesh displaced via EMUM (Fig. 9b). At this stage, the deformation remains small enough to preserve overall element quality and enable a qualitatively reliable hemodynamics prediction. The most visible mesh distortion occurs in the boundary layer region near the capped ends of the artery segment (black circle), where the boundary layers appear squeezed and slightly perturbed. While the elements remain untangled, some distortion and elongation is observed in regions adjacent to the stent crown (red rectangle), where the local deformation is more pronounced. However, due to the fine mesh resolution in these areas, no numerical instabilities are observed. This early-stage ISR growth between stent struts – without accounting for endothelial coverage – is considered acceptable for short-term predictions (e.g., up to $50$ days). However, for later stages (several months post-PCI), this modeling assumption becomes physiologically unrealistic, particularly in terms of its influence on lumen deformation. We expand on this limitation in the Discussion.

## Discussion

In this work, we introduce a coupled fluid-solid model of coronary arteries with in-stent restenosis in the presence of a drug-eluting stent. The scheme summarized in Fig. 1a describes the main physics of ISR, hemodynamics, and DESs with hydrophobic drug, along with their interaction at the lumen-wall interface $\Gamma _{b,w}$ and stent surface $\Gamma _s$. To address the multiscale nature of these phenomena in time, we implement a quasi-rigid lumen deformation strategy and a quasi-steady approach that efficiently homogenizes hemodynamic-related quantities.

We use the test case of an idealized artery with a one-ring stent, sketched in Fig. 1b, c, to verify the quasi-rigid and quasi-steady approximations (see Fig. 3). The quasi-steady assumption implies that, once the periodic regime is established, flow conditions remain stable for an entire day. The quasi-rigid approximation assumes that the lumen-wall interaction does not significantly change within the time scale of one day. Testing the pulsatile regime shows that the time-averaged concentration $c_b$ coincides with the value obtained using a steady blood velocity $\overline{\varvec{u}}$. Moreover, the time-averaged hemodynamic indicators are well-approximated by their steady-state counterparts. Regarding the influence of the ISR-induced lumen deformation on the quantities in $\Omega _b$, Fig. 4a, b indicate that the ISR displacement notably affects blood velocity, streamlines, and $\text{WSS}$. In the quasi-rigid approach, the EMUM technique has the practical advantage of avoiding remeshing and preserving mesh nodes mapping at the fluid-solid interface $\Gamma _{b,w}$, reducing computational overhead. When focusing solely on drug concentration $c_b$, the wall displacement has a limited impact and can be omitted if drug release into the bloodstream is the primary focus (see Fig. 4c). Here, we model hydrophobic drug by applying tailored Neumann-Robin boundary conditions at the lumen-wall interface $\Gamma _{b,w}$. Accounting for a one-way flux of $c_b$ into the arterial wall does not greatly alter the magnitude of the overall drug concentration $c_w$ or ISR prediction. However, it produces an asymmetry in both $c_w$ and $\vert \varvec{d}_{\text {ISR}} \vert$ around the ring stent that a standalone solid model cannot replicate (see Figs. 5a and 6b). We further observe in Fig. 5b – d that drug within the fluid domain $\Omega _b$ is largely washed out, concentrating primarily at the lumen-wall interface $\Gamma _{b,w}$. The main effect on ISR growth comes from the hemodynamic indicators. In particular, we observe that $\text{WSS}$, implemented through the shape index SI, has the strongest influence on neointimal thickness. This also justifies bypassing the more expensive unsteady simulations needed for the computation of $\text{OSI}$ in favor of a quasi-steady approach. The influence of coupled hemodynamics is evident both in the neointimal thickness $90$ days after PCI and in its evolution in time (see Fig. 6a, c, respectively). Even after the EC density returns to equilibrium, non-physiological flow conditions (e.g., low $\text{WSS}$ or high $\text{OSI}$) can still promote neointimal growth. Thus, ISR may continue to increase after $90$ days, despite the near-complete restoration of the EC layer.

After verifying our assumptions and identifying the most influential factors, we apply the model to a more realistic, patient-specific setting. The importance of this test case builds on evidence that anatomical accuracy, including artery curvature, significantly affects hemodynamics and drug distribution^49^. We try to move closer to a clinically relevant workflow, as exemplified in Fig. 7: we extract data from OCT images of a pre-stented artery (see Fig. 2), virtually implant a single-crown stent for demonstration, and then perform the simulation on the fluid-solid mesh with matching lumen-wall interface, shown in Fig. 1d. The results obtained from the complete fluid-solid model, reported in Fig. 8, align well with the expectations from the one-ring stent case and demonstrate the suitability of this coupling framework for complex, patient-specific scenarios.

Despite these first promising results, several limitations remain to be addressed:

- Computational costs. Because of the hybrid parallelization approach (multi-threading in FEAP and multi-core in XNS), the simulations are restricted to a single computational node with a maximum of 96 threads and 96 cores. This constraint contributes to the high computational cost observed for the patient-specific case. Future work should explore algorithmic optimization and potentially apply model reduction techniques to improve efficiency.
- Refinements of the fluid model. Allowing non-matching interfaces could increase accuracy in the fluid domain, especially in the computation of hemodynamic indicators. Furthermore, the effect of more complex Windkessel boundary conditions, pulsatile artery response to blood pressure and ventricle movement^87^ should be tested. Although the stented region is substantially stiffened and largely non-deformable, the artery adjacent to the stent may still respond to blood pressure. Due to the high density of SMCs in the vessel wall, coronary arteries are often modeled as rigid in the literature, also before stenting. Nonetheless, it would be worthwhile to investigate coronary compliance in future studies to assess its implications in this modeling framework. Previous findings indicate that arterial wall pulsation can significantly influence hemodynamic indicators^37^, though further investigation is needed to determine whether this effect meaningfully impacts cell dynamics and, ultimately, ISR growth. An adaptive time-stepping scheme, particularly in the early days of drug elution could further enhance precision. A more accurate prediction could be achieved with a subcycling approach, where the fluid computation is performed every $N_b$-portion of each daily iteration, i.e., $\frac{\Delta t_w}{N_b}$, together with step-wise extrapolation of drug release and ISR. Nevertheless, the lumen deformation is not significantly altered over a daily iteration, as demonstrated in Fig. 3a.
- Patient-specific features and parameter tuning. Currently, stent indentation and plaque effects are neglected in the virtual stent implantation. Including these factors, along with patient-specific calibrations (e.g., permeability, diffusion coefficients, plaque type), could improve the model fidelity. More realistic testing, and potential experimental validation, should involve at least a two-crown stent in the virtual stenting approach, given the altered stress response of a single-crown design. Moreover, using shorter-crown stents could provide higher radial strength, making the simulations more comparable to clinical scenarios. At this stage, however, the single-crown setup mainly serves to demonstrate the model capacity to handle more complex configurations, rather than to replicate an exact clinical case.
- Realistic ISR growth and endothelial coverage. While EMUM eliminates the need for remeshing, it also restricts the deformation at the fluid-solid interface, preventing overlapping of the stent, lumen, and wall. A key limitation is its reduced robustness under large deformations, which may result in mesh element distortion or even tangling near geometrically complex regions such as stent crowns. At this stage, our setup can only capture ISR growth between stent struts and does not account for endothelial coverage of the stent surface or subsequent neointimal thickening over the struts. While this simplification is acceptable for short-term predictions (e.g., up to $50$ days), it becomes unphysiological for modeling longer-term ISR progression (e.g., beyond $6$ months). As a consequence, the lumen deformation and resulting hemodynamics do not fully reflect the ISR patterns observed in vivo. Large deformations or a gradual endothelial cover of the stent would benefit from a remeshing strategy, an immersed-boundary/level-set approach, or more advanced techniques that support large deformations while preserving mesh quality^88^. These considerations also tie into existing constraints in modeling ISR growth on the solid side.

The fourth limitation needs be addressed to ensure physiologically meaningful ISR prediction over longer time horizons, especially for time spans up to a year (standard in clinical follow-ups). The current method restricts the amount of ISR growth that can be modeled because it employs trilinear interpolations of the displacement field. Hexahedral elements are associated with volumetric locking due to the incompressibility of soft tissues as well as shear locking in bending-dominated scenarios^89,90^, causing the tissue to behave more stiffly than it would physiologically. Consequently, accurately reproducing the full coverage of stent struts with neointima becomes challenging. Addressing this issue requires incorporating advanced methods to capture boundary displacements with topological changes. These strategies would mitigate locking effects and allow more realistic wall deformation under ISR, while only moderately increasing the computational cost of the solid model – compared to other computationally intensive components of the overall framework. This limitation is technical in nature and can be addressed through established numerical strategies. The most critical barrier to advancing towards clinical applicability lies in the third limitation. Specifically, robust validation against medical data, parameters calibration through experiments, and an extension to a comprehensive patient-specific setup – including both realistic artery geometries and commercial stent designs – are essential next steps.

The authors intend to address many of these limitations in the future work. We plan to optimize the computational framework to improve efficiency and streamline both pre-processing and post-processing, aiming to achieve a more clinically oriented workflow. We also plan to conduct a thorough sensitivity analysis, examining time-step sizes, coupling steps, and patient-specific parameter tuning. To overcome element locking, we will employ a level-set approach to track the expanding neointima with an embedded interface, enabling physiological coverage of the stent surface and consequent update of the fluid domain deformation. The boundary conditions on the stent-tissue and lumen-tissue interfaces can then be enforced weakly using the Nitsche method^91,92^.

The primary objective of this study is to propose a coupled multiphysics framework that integrates a continuum model of cell species in the arterial wall, the pharmacokinetics of both wall and luminal drug release, hemodynamics, and an ISR growth model. Although these components have been investigated in prior work, they are typically addressed separately or via hybrid agent-based models of cell dynamics, which are limited to two-dimensional settings. To the best of our knowledge, no existing approach integrates all these processes into a three-dimensional framework using continuum-based models for all fluid and solid components, especially in patient-specific scenarios. This work offers potential clinical relevance by enabling prediction of ISR outcomes for different stent types, designs and implantation techniques, including parameters such as balloon pressure and indentation. Additionally, it could facilitate tailored, patient-specific drug placement on stents to optimize arterial healing through different drug loading configurations.

## Data Availability

The datasets generated and/or analysed during the current study are available from the corresponding author on reasonable request. BeamMe^84^ and 4C^85^ are both open source. The in-house code XNS will be made open-access soon. The software package FEAP is proprietary and can therefore not be made available. The custom-written FEAP routines and Python coupling scripts are stored on GitLab, access can be granted from the corresponding author on reasonable request.

## Footnotes

1 Integrating Eq. (6) over $\Delta t_w$ yields $\displaystyle\frac{1}{\Delta t_w}\int _{t^{(n)}_{-}}^{t^{(n)}_{+}} \frac{\partial c_b(t) dt }{\partial t} - \frac{1}{\Delta t_w}\int _{t^{(n)}_{-}}^{t^{(n)}_{+}} D_b \Delta c_b (t) dt + \frac{1}{\Delta t_w}\int _{t^{(n)}_{-}}^{t^{(n)}_{+}} \varvec{u}(t) \cdot \nabla c_b(t) dt = 0$. Under the above assumption, the first term drops out and, assuming negligible temporal variation of $c_b$ over $\Delta t_w$, the remaining terms can be written as $\displaystyle\frac{1}{\Delta t_w}\int _{t^{(n)}_{-}}^{t^{(n)}_{+}} \bullet ~dt = \frac{N_{HB}}{\Delta t_w} \int _{\Delta T_{HB}} \bullet ~dt$, where $N_{HB}$ corresponds to the number of cardiac cycles $\Delta T_{HB}$ within $\Delta t_w$. Additionally, $\displaystyle\frac{N_{HB}}{\Delta t_w} \int _{\Delta T_{HB}} \varvec{u}(t) dt \approx \frac{N_{HB} T_{HB}}{\Delta t_w}\overline{\varvec{u}} = \overline{\varvec{u}}$ corresponds to the blood velocity in a steady regime.
